# Brain-like variational inference

**Published:** 2025-05-16

**Authors:** Hadi Vafaii, Dekel Galor, Jacob L. Yates

**Affiliations:** 1 UC Berkeley

## Abstract

Inference in both brains and machines can be formalized by optimizing a shared objective: maximizing the evidence lower bound (ELBO) in machine learning, or minimizing variational free energy (F) in neuroscience (ELBO=−F). While this equivalence suggests a unifying framework, it leaves open how inference is implemented in neural systems. Here, we show that online natural gradient descent on F, under Poisson assumptions, leads to a recurrent spiking neural network that performs variational inference via membrane potential dynamics. The resulting model—the iterative Poisson variational autoencoder (iP-VAE)— replaces the encoder network with local updates derived from natural gradient descent on F. Theoretically, iP-VAE yields a number of desirable features such as emergent normalization via lateral competition, and hardware-efficient integer spike count representations. Empirically, iP-VAE outperforms both standard VAEs and Gaussian-based predictive coding models in sparsity, reconstruction, and biological plausibility. iP-VAE also exhibits strong generalization to out-of-distribution inputs, exceeding hybrid iterative-amortized VAEs. These results demonstrate how deriving inference algorithms from first principles can yield concrete architectures that are simultaneously biologically plausible and empirically effective.

## 1 Introduction

Artificial intelligence and theoretical neuroscience share a foundational principle: understanding the world requires inferring hidden causes behind sensory observations (early work: [1–3]; modern interpretations: [4–9]). This principle is formalized through Bayesian posterior inference [10–12], with *variational inference* [4, 13–17] offering an optimization-based approximation. Variational inference employs an identical objective function across both fields, albeit under different names. In machine learning, it is called *evidence lower bound* (ELBO), which forms the basis of variational autoencoders (VAEs; [18–21]); whereas, neuroscience refers to it as *variational free energy* (F), which subsumes predictive coding [22, 23] and is presented as a unified theory of brain function [24]. ELBO is exactly equal to negative free energy (ELBO=−F; [25]), and this mathematical equivalence offers a powerful common language for describing both artificial and biological intelligence [26].

Despite this promising connection, neither ELBO nor F have proven particularly prescriptive for developing specific algorithms or architectures [27, 28]. Instead, the pattern often works in reverse: effective methods are discovered empirically, then later recognized as instances of these principles. In machine learning, diffusion models were originally motivated by non-equilibrium statistical mechanics [29], only later understood as a form of ELBO maximization [30, 31]. Similarly, in neuroscience, *predictive coding* (PC; [22]) was first proposed as a heuristic model for minimizing prediction errors [32], before being reinterpreted as F minimization [23, 33]. This pattern of post-hoc theoretical justification, rather than theory-driven derivation, is common across both disciplines.

Then why pursue a general theory at all? A unifying framework offers several advantages: it clarifies the design choices underlying successful models, explains why they work, and guides the development of new models. The recent *Bayesian learning rule* (BLR; [34]) exemplifies this potential. It re-interprets a broad class of learning algorithms as instances of variational inference, optimized via natural gradient descent [35] over approximate posteriors $q_{\lambda }$. By varying the form of $q_{\lambda }$ and the associated approximations, BLR not only recovers a wide range of algorithms—from SGD and Adam to Dropout—but, crucially, it also offers a principled recipe for designing new ones [34]. Thus, BLR transforms a post-hoc theoretical justification into a prescriptive framework for algorithm design.

To summarize, the essential prescriptive choices are specifying $q_{\lambda }$ and deciding how to optimize the variational parameters, λ. In recent years, the machine learning community has largely embraced *amortized inference* [18, 19, 36–38], where a neural network is trained to produce λ in a single forward pass for each input sample. In contrast, neuroscience models have traditionally favored *iterative inference* methods [22, 39], which align more naturally with the recurrent [40–44] and adaptive [45–49] processing observed in cortical circuits (but see Gershman and Goodman [36] for a counterpoint). Despite this fundamental divide in methodology, systematic comparisons between iterative and amortized inference variants remain scarce, particularly when evaluated against biologically relevant metrics.

In this paper, we introduce *F**ree energy* *O**nline* *N**atural-gradient* *D**ynamics* (FOND), a prescriptive framework for deriving brain-like inference dynamics from F minimization. We show that natural gradient descent on F results in a family of iterative VAE architectures with distinct computational and biological advantages. The paper is organized as follows:

- In section 2 and the associated appendix A, we review and synthesize existing models from machine learning and neuroscience, showing how they can be unified under the variational inference framework via specific choices of distributions and inference methods (Fig. 2).
- In section 3 and the associated appendix B, we derive neural dynamics as natural gradient descent on free energy. This yields a novel family of iterative VAEs, including a spiking variant—the iterative Poisson VAE (iP-VAE)—which performs online Bayesian inference through membrane potential dynamics, with emergent lateral competition and normalization.
- In section 4, we show that iterative inference consistently outperforms amortized methods, with iP-VAE achieving the best reconstruction-sparsity trade-off, while using 25× fewer parameters and integer-valued spike counts. Appendix C shows that iP-VAE exhibits (i) emergent cortical response properties, and (ii) improved out-of-distribution generalization.
- In section 5, we relate our results to advances in sequence modeling, and discuss how expressive nonlinearities, emergent normalization, and effective stochastic depth contribute to iP-VAE’s computational strengths.

## 2 Background and Related Work

### Notation and conventions.

We start with a generative model that assigns probabilistic beliefs to observations, $x\in {\mathbb{R}}^{M}$ (e.g., images), through invoking K-dimensional latent variables, $z:p_{\theta }(x)=\int p_{\theta }(x,z)dz=\int p_{\theta }(x|z)p_{\theta }(z)dz$, where θ are its adaptable parameters (e.g., neural network weights or synaptic connection strengths in the brain). Throughout this work, we color-code the generative and inference model components as blue and red, respectively.

### Variational inference and the ELBO objective.

Following the *perception-as-inference* framework [1, 2], we formalize perception as Bayesian posterior inference. In appendix A.1, we provide a historical background, and in appendix A.2, we review the challenges associated with posterior inference. We overcome these challenges by approximating the true but often intractable posterior, $p_{\theta }(z|x)$, using another distribution, $q_{\phi }(z|x)$, referred to as the *approximate* (or *variational*) posterior. In appendix A.3, we provide more details about how *variational inference* [15, 17] approximates the inference process, and derive the standard *E**vidence* *L**ower* *BO**und* (ELBO) objective [13, 15, 17]:

(1) $$\underset{\text{model evidence}}{\underset{︸}{\log p_{\theta }(x)}}=\underset{\text{ELBO}(x;\theta ,\phi )}{\underset{︸}{E_{z∼q_{\phi }(z|x)}\left[\log\frac{p_{\theta }(x,z)}{q_{\phi }(z|x)}\right]}}+\underset{\text{Kullback-Leibler (KL) divergence}}{\underset{︸}{D_{\text{KL}}\left(q_{\phi }(z|x)\|p_{\theta }(z|x)\right)}}.$$

Importantly, maximizing the ELBO minimizes the intractable Kullback-Leibler (KL) divergence between the approximate and true posterior. This can be seen by taking gradients of eq. (1) with respect to ϕ. The model evidence on the left-hand side does not depend on ϕ, making the gradients of the two terms on the right additive inverses. Thus, ELBO maximization directly enhances the quality of posterior inference. In theoretical neuroscience, the negative ELBO is referred to as variational free energy (F), which is the mathematical quantity central to the *free energy principle* [24, 25].

In the remainder of this section, we demonstrate how diverse models across machine learning and neuroscience emerge as instances of F minimization through two fundamental design choices:

1. **Choice of distributions** (appendix A.4):
  - approximate posterior $q_{\phi }(z|x)$, (ii) prior $p_{\theta }(z)$, and (iii) likelihood $p_{\theta }(x|z)$
2. **Choice of inference method** (appendix A.8):
  - *amortized* (e.g., learned neural network) vs. (ii) *iterative* (e.g., gradient descent)

### Variational Autoencoder (VAE) model family.

Variational Autoencoders (VAEs) transform the abstract ELBO objective into practical deep learning architectures [18–20]. The standard Gaussian VAE (G-VAE) exemplifies this approach by assuming factorized Gaussian distributions for all three distributions, with the approximate posterior $q_{\phi }(z|x)$ implemented as a neural network that maps each input x to posterior parameters: $\text{enc}(x;\phi )\to \left(\mu (x),\sigma^{2}(x)\right)$. This *amortization* of inference—using a single network to approximate posteriors across the entire dataset—is a defining characteristic of VAEs. Alternative distribution choices are also possible; for instance, replacing both prior and posterior with Poisson distributions yields the Poisson VAE (P-VAE; [50]), which better aligns with neural spike-count statistics [51–53]. We derive the VAE loss in appendix A.5, and discuss both G-VAE and P-VAE extensively in appendices A.6 and A.7.

### Sparse coding and predictive coding as variational inference.

Two major cornerstones of theoretical neuroscience, sparse coding (SC; [54]) and predictive coding (PC; [22]), can also be derived as instances of ELBO maximization (or equivalently, F minimization), given specific distributional choices [33, 55, 56]. SC and PC share two key characteristics that distinguish them from standard VAEs. First, they both use a Dirac-delta distribution for the approximate posterior, effectively collapsing it to a point estimate. But they differ in their prior assumptions: PC employs a Gaussian prior, while SC uses a sparsity-promoting prior (e.g., Laplace; Fig. 2). Second, instead of amortized inference as in VAEs, both PC and SC employ iterative inference, better aligning with the recurrent nature of neural computation [40–44]. See appendix A.8 for an in-depth comparison.

In appendix A.9, we provide a pedagogical derivation of the Rao and Ballard [22] objective for linear PC. Specifically, we show that minimizing free energy with the aforementioned assumptions yields the following objective: $F\left(x;\text{Φ},\mu_{0},\mu \right)=\frac{1}{2}\left[{\left(x-\text{Φ}\mu \right)}^{2}+{\left(\mu -\mu_{0}\right)}^{2}\right]$, where $x\in {\mathbb{R}}^{M}$ are stimuli, $\mu \in {\mathbb{R}}^{K}$ are neural activations, and $\text{Φ}\in {\mathbb{R}}^{M\times K}$ is the linear decoder (or “dictionary” in sparse coding). PC assumes neural activity μ evolves according to $\dot{\mu }=-\nabla \mu F$, resulting in:

(2) $$\dot{\mu }=\underset{\text{feedforward drive}}{\underset{︸}{{\text{Φ}}^{T}x}}\;-\;\underset{\text{lateral connections}}{\underset{︸}{{\text{Φ}}^{T}\text{Φ}\mu }}\;-\;\underset{\text{leak term}}{\underset{︸}{\left(\mu -\mu_{0}\right)}}.$$

### Online inference through natural gradient descent.

In a recent landmark paper, Khan and Rue [34] proposed the *Bayesian learning rule* (BLR), unifying seemingly disparate learning algorithms as instances of variational inference. Different algorithms arise from different variational posteriors and approximation choices, optimized via natural gradient descent [35, 57] on a Bayesian objective which reduces to the ELBO when the likelihood is known [58, 59]. BLR demonstrates the utility of a unifying framework, because it relates seemingly different algorithms through the same lens, allowing us to better understand their characteristics and the reason for their success. But also, critically, BLR provides a recipe for deriving new algorithms from first principles [34].

Building on BLR [34], Jones et al. [60] proposed the *Bayesian online natural gradient* (BONG), a sequential inference method that performs a single natural gradient update on the expected log-likelihood, initialized at the prior predictive distribution. BONG recovers exact Bayesian inference if the model is conjugate, and it performs well empirically, even with a simple rolling update scheme in which the posterior at each step becomes the prior for the next (Fig. 7).

### Our contributions.

In this section so far, along with the corresponding appendix A, we reviewed how seemingly different models across machine learning and neuroscience can be understood as instances of F minimization (Fig. 2). The differences among them arise from the choices we make on the distributions and the inference methods (appendix A.10).

**Figure 1:**
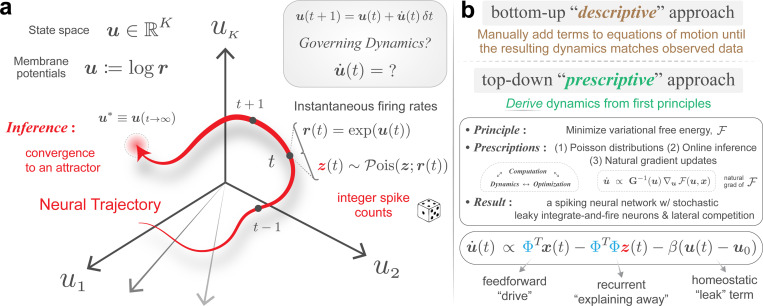
Inferential and dynamical accounts of perception are unified under variational inference. **(a)** Perception is framed as a dynamical process of convergence to attractors in a neural state space, where membrane potentials evolve and generate spikes along the way. **(b)** Our prescriptive approach derives neural dynamics by minimizing free energy via natural gradient descent, yielding a spiking network with lateral competition. The resulting architectures are principled and empirically effective. Code, data, and model checkpoints are available here: github.com/hadivafaii/IterativeVAE

Building on the unification potential shown above, we introduce *F**ree energy* *O**nline* *N**atural-gradient* *D**ynamics* (FOND): a framework that combines online Bayesian inference with natural gradient updates to derive brain-inspired adaptive inference algorithms from F minimization. Specifically, FOND provides a principled framework for deriving neural dynamics by first explicitly identifying dynamical variables (Fig. 1a), and then formulating their temporal evolution as natural gradient descent on variational free energy (Fig. 1b), with an online update scheme for real-time adaptation to incoming sensory data (Fig. 7).

To demonstrate the utility of FOND, we apply it to derive a new family of iterative VAE models (iP-VAE, iG-VAE, $\text{i}G_{\varphi }$-VAE; Table 1) that combine the probabilistic foundation of VAEs with brain-inspired iterative dynamics. Unlike previous post-hoc interpretations, FOND adopts a prescriptive approach: it starts from theoretical principles and derives architectures in a top-down manner, making explicit the connection between concrete design choices and the resulting computational properties.

In what follows, we present the theoretical derivations underlying these architectures, evaluate their empirical performance, and discuss their implications for both neuroscience and machine learning.

## 3 Theory: Deriving a Spiking Inference Model from Variational Principles

In this section, we apply the FOND framework to derive brain-like inference dynamics from first principles. We begin by identifying the key prescriptive choices required for deriving these dynamics. We then introduce Poisson assumptions and derive membrane potential updates as natural gradient descent on variational free energy (F), leading to the iterative Poisson VAE (iP-VAE) architecture.

### Three distributional choices.

To fully specify F, we must choose an approximate posterior, a prior, and a conditional likelihood. We use Poisson distributions for both posterior and prior, as it leads to more brain-like integer-valued spike count representations (see appendix B.1 for a discussion). Later in the appendix, we show how this derivation extends to Gaussian posteriors with optional nonlinearities applied after sampling, illustrating the generality and flexibility of FOND.

For the likelihood, we assume factorized Gaussians throughout this work (a standard modeling choice in both sparse coding [54] and predictive coding [22]). In the main text, we focus on linear decoders, $p_{\theta }(x|z)=N(x;\text{Φ}z,\text{I})$, where Φ is commonly referred to as the *dictionary* in sparse coding literature. In the appendix, we show how our results naturally extend to nonlinear decoders with learned variance [61]; and we explore these extensions both theoretically and empirically.

### Prescriptive choices.

To derive dynamics from first principles, we must address two critical questions: what are the dynamical variables, and what equations govern their time evolution? See appendix B.2. The key prescription in FOND is that once the dynamic variables are specified, their dynamics are governed by natural gradient descent on F.

For a neurally plausible model, we prescribe the dynamic variables to be real-valued membrane potentials, $u\in {\mathbb{R}}^{K}$, where K is the number of neurons. To generate spike counts from u, we assume the canonical Poisson parameterization, defining firing rates as r:=exp(u) (Fig. 1a). This choice is both mathematically convenient, since it avoids constrained optimization over a strictly positive variable, and biologically motivated, as the spiking threshold of real neurons is well-approximated by expansive nonlinearities [62, 63].

Under the assumption that dynamics, computation, and optimization are three expressions of the same underlying *process* [64–67] (i.e., inference⇔dynamics), membrane potentials evolve to minimize variational free energy (F). To respect the curved geometry of distribution space, this optimization is implemented via natural gradient descent [34, 57, 68] (Fig. 1b).

### Poisson variational free energy.

Given a Poisson posterior and prior, and a factorized Gaussian likelihood with a linear decoder, the free energy (negative ELBO from eq. (1)) takes the form:

(3) $$F\left(x;\text{Φ},u_{0},u\right)=\underset{L_{\text{recon}.}:\;\text{reconstruction term (}distortion\text{)}}{\underset{︸}{E_{z∼q_{u}(z|x)}\left[\frac{1}{2}\|x-\text{Φ}z{\|}_{2}^{2}\right]}}+\beta \underset{L_{\text{KL}}:\;\text{KL term (}coding\;rate\text{)}}{\underset{︸}{{\sum_{i=1}^{K}\left(e^{u}⊙\left(u-u_{0}\right)-\left(e^{u}-e^{u_{0}}\right)\right)}_{i}}},$$

where $u_{0}\in {\mathbb{R}}^{K}$ and $u\in {\mathbb{R}}^{K}$ are the prior and posterior membrane potentials, K is the latent dimensionality, ⊙ represents the element-wise (Hadamard) product, and β is a positive coefficient that controls the *rate-distortion* trade-off [69, 70]. See appendix B.3 for a detailed derivation.

Next, we compute the gradient of F with respect to the variational parameters, i.e., $\nabla_{u}F$.

### Free energy gradient: the reconstruction term.

$L_{\text{recon}.}$ is the expected value of the prediction errors under the approximate posterior. Since this expectation is intractable in general, we follow standard practice in variational inference and approximate it using a single Monte Carlo sample [34, 60]: $L_{\text{recon}.}\approx \frac{1}{2}\|x-\text{Φ}z(u){\|}_{2}^{2}$, where $z(u)∼q_{u}(z|x)$.

Since the reconstruction loss depends on the sample z, which is a stochastic function of the firing rate r=exp(u), we propagate gradients through both the exponential nonlinearity and the sampling process (i.e., $u\to \text{r}\to z\to L_{\text{recon}.}$). We apply the chain rule twice to get:

(4) $$\nabla_{u}L_{\text{recon}.}=\frac{\partial \text{r}}{\partial u}\frac{\partial z}{\partial \text{r}}\frac{\partial }{\partial z}L_{\text{recon}.}\approx e^{u}⊙\frac{\partial z}{\partial \text{r}}⊙\left(-{\text{Φ}}^{T}(x-\text{Φ}z(u))\right).$$

We further simplify the expression by applying the straight-through gradient estimator [71], treating ∂z/∂r≈I for the purpose of deriving inference equations. During model training, however, we update the generative model parameters by utilizing the Poisson reparameterization algorithm [50]. This straight-through approximation of the sample gradient yields:

(5) $$\nabla_{u}L_{\text{recon}.}\approx e^{u}⊙\left(-{\text{Φ}}^{T}(x-\text{Φ}z(u))\right).$$

### Free energy gradient.

The KL-term gradient easily follows from eq. (3): $\nabla_{u}L_{\text{KL}}=e^{u}⊙\left(u-u_{0}\right)$. Combine this KL-term gradient with the reconstruction-term gradient from eq. (5) to get:

(6) $$\nabla_{u}F\left(x;\text{Φ},u_{0},u\right)\approx e^{u}⊙\left[-{\text{Φ}}^{T}(x-\text{Φ}z(u))+\beta \left(u-u_{0}\right)\right].$$

### Natural gradients: applying Fisher preconditioning.

FOND prescribes that neural dynamics follow natural gradients of the free energy, $\dot{u}:=-{\text{G}}^{-1}(u)\nabla_{u}F$, where G is the Fisher information matrix. For Poisson distributions in the canonical form, we have: G(u)=exp(u) (see appendix B.4 for a derivation). Combining this result with eq. (6) yields:

(7) u˙∝ΦTx︸feedforward “drive”−ΦTΦz(u)︸recurrent “explaining away”−βu−u0︸homeostatic “leak” term

This concludes the derivation of our core theoretical result underlying the iP-VAE architecture. Natural gradient descent on F yields circuit dynamics that share important characteristics with neural models developed since the early 1970s [72–75], while offering a notable biological advantage over standard predictive coding (PC). Namely, recurrent interactions in iP-VAE (eq. (7)) occur through discrete spikes z, rather than continuous membrane potentials as in PC (eq. (2)), better aligning with how real neurons communicate [76]. In appendix B.5, we interpret each term in the dynamics and explain the rationale behind its naming. In appendix B.6, we extend the derivation to nonlinear decoders; and in appendix B.7, we extend to Gaussian posteriors with optional nonlinearities. Finally, appendix B.8 explores this biological distinction with standard PC in more detail.

### Online inference: adding time.

Perception is an ongoing process that requires continually updating beliefs as data arrives, rather than reverting to a fixed prior in the distant past. To model this sequential process, we adopt a rolling update scheme similar to BONG [60], where the current posterior becomes the next prior (Fig. 7). Combining this online structure with the dynamics from eq. (7), we arrive at the following discrete-time update rule:

(8) $$u_{t+1}=u_{t}+{\text{Φ}}^{T}x-{\text{Φ}}^{T}\text{Φ}z_{t},$$

where $u_{t}$ and $u_{t+1}$ are interpreted as the prior and posterior membrane potentials at time t (Fig. 5).

Notably, the leak term in eq. (7)—which stems from the KL term in eq. (3)—disappears in this discrete-time update. This is because we perform only a single step per time point, and no longer assume a static prior, but one that evolves continuously over time (Fig. 7). In appendix B.9, we derive the general form of free energy for sequences, and in appendix B.10, we explain why the KL contribution vanishes in the single-update limit.

### Lateral competition as a stabilizing mechanism.

The recurrent connectivity matrix, $W:={\text{Φ}}^{T}\text{Φ}$, has a stabilizing effect. To demonstrate this, we transform the discrete-time dynamics in eq. (8) from membrane potentials to firing rates, yielding the following multiplicative update for neuron i:

(9) $${\text{r}}_{t+1,i}={\text{r}}_{t,i}\frac{\exp{\left({\text{Φ}}^{T}x\right)}_{i}}{\exp\left(W_{ii}z_{t,i}\right)\prod_{j=1,j\neq i}^{K}\exp\left(W_{ij}z_{t,j}\right)}.$$

where ${\text{r}}_{t}=\exp\left(u_{t}\right)$ are firing rates, and $z_{t}∼P\text{ois}\left(z;{\text{r}}_{t}\right)$ are sampled spikes. The resulting denominator reveals a form of multiplicative divisive normalization: co-active neurons suppress each other proportionally to their spike output.

As seen in eq. (9), the recurrent term $Wz_{t}$ acts as a stabilizing force in two ways. First, its diagonal entries are positive (i.e., $W_{ii}={\left\|{\text{Φ}}_{\cdot i}\right\|}_{2}^{2}>0$); therefore, neurons that spike strongly receive self-suppression, dampening activity at the next time point. Second, the off-diagonal entries couple neurons with overlapping tuning. Due to this coupling, vigorously active neurons extend suppressive influence toward their similarly-tuned neighbors (i.e., when $W_{ij}>0$), preventing excess activity. In addition to stabilization, such competitive interactions also underlie the sparsification dynamics observed in cortical circuits [77–79], and are widely regarded as a hallmark of sparse coding [39, 80].

In sum, we have shown that natural gradient descent on free energy, combined with biologically motivated assumptions, leads to principled and interpretable neural dynamics. As our next step, we apply the theoretical derivations (eq. (7), appendix B.7, and Fig. 5) to design specific instances of iterative VAEs within the broader FOND framework. These include: the iterative Poisson VAE (iP-VAE), the iterative Gaussian VAE (iG-VAE), and the iterative Gaussian-relu VAE ($\text{i}G_{\text{relu}}$-VAE).

## 4 Experiments

We evaluate the family of iterative VAE models introduced in this work (iP-VAE, iG-VAE, $\text{i}G_{\text{relu}}$-VAE; Table 1). These models share identical inference dynamics derived in section 3 and appendix B.7, differing only in their latent variable distributions (Poisson vs. Gaussian) and an optional nonlinearity (e.g., relu [81]) applied after sampling from the posterior. This systematic comparison helps isolate the relative influence of each component on learning brain-like representations that generalize. To evaluate the impact of inference methods, we compare these iterative models to their amortized counterparts (P-VAE, G-VAE, $G_{\text{relu}}$-VAE), all implemented with identical convolutional encoders.

**Figure 2:**
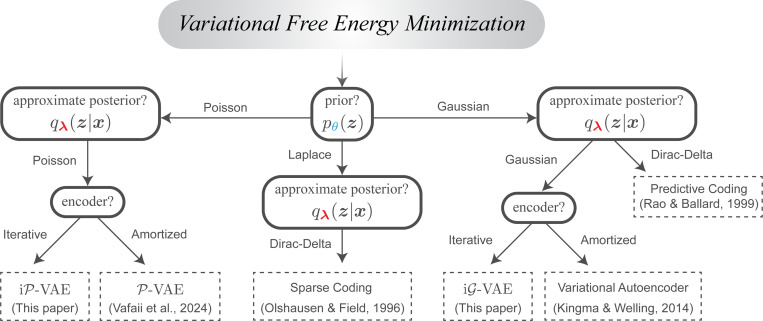
A wide range of models across machine learning and theoretical neuroscience can be unified under variational free energy $\left(F\right)$ minimization. Each model is derived from the same source, F minimization, but with distinct prescriptive choices that define and distinguish the model (appendix A.10). Motivated by this unification potential, we introduce FOND, a framework for deriving brain-like inference algorithms from first principles. Specifically, FOND prescribes that natural gradient descent on F determines neural inference dynamics. We apply FOND to derive a family of iterative VAE architectures, including the spiking iP-VAE, that combine the probabilistic foundations of VAEs with the biologically plausible iterative inference characteristic of neuroscience models. See Table 1 for a complete summary.

We also compare to classic normative models in neuroscience, including two predictive coding models, standard PC [22] and incremental PC (iPC; [82]), and the locally competitive algorithm (LCA; [39]), a sparse coding model that can be viewed as a deterministic, non-spiking precursor to iP-VAE. See Table 1 for a summary of models, and Fig. 2 for a visualization of the model tree. Additional comparisons to hybrid iterative-amortized VAEs [83, 84] are presented in the appendix.

### Learning to infer.

To learn the model parameters, we use a training scheme that accumulates gradients across the entire inference trajectory and applies a single update at the end. Specifically, we perform $T_{\text{train}}$ inference steps per input batch and optimize model weights using the accumulated gradients across all steps (not just the final state). This procedure is analogous to backpropagation through time [85, 86], and $T_{\text{train}}$ can be interpreted as the effective model “depth” [43, 87–89] (Fig. 5). We find that the choice of $T_{\text{train}}$ has a significant effect on quantitative metrics, making it an important hyperparameter to track. We explore various $T_{\text{train}}$, but report performance using the same $T_{\text{test}}=1,000$ steps.

### Hyperparameters.

In addition to $T_{\text{train}}$, we explore various β values [70], which controls the reconstruction-KL trade-off (eq. (3)). For iP-VAE and P-VAE [50], varying β traces out a reconstruction-sparsity landscape, loosely analogous to a rate-distortion trade-off [69]. All models have a latent dimensionality of K=512, unless specified otherwise. In the main paper, we stick to models with linear decoders comprised of a single dictionary, $\text{Φ}\in {\mathbb{R}}^{M\times K}$, where M is the input dimensionality (i.e., number of pixels). We explore more general nonlinear decoder architectures in the appendix. See appendix C.1 for additional implementation, hyperparameter, and training details.

### Tasks and Datasets.

We evaluate models in several ways, including convergence behavior, reconstruction–sparsity trade-off, downstream classification, and out-of-distribution (OOD; [90, 91]) generalization. We train and evaluate models on two datasets: (1) whitened 16 × 16 natural image patches from the van Hateren dataset [92], used to assess convergence and reconstruction–sparsity trade-offs; (2) MNIST [93], used for reconstruction, classification, and OOD generalization tests. A good portion of these results, including the OOD ones, are presented in the appendix, as our primary focus in this paper is theoretical, and these results warrant their focused exploration in future work.

### Performance metrics.

We define model *representations* as samples drawn from the posterior at each time point (e.g., z(t) in Fig. 1a), and quantify performance using the following metrics. For reconstruction, we compute the coefficient of determination $\left(R^{2}\right)$ between the input image, x, and its reconstruction, $\hat{x}=\text{Φ}z$. $R^{2}(x,\hat{x})$ quantifies the proportion of input variance explained and yields a bounded, dimensionality-independent score in [0, 1]. For sparsity, we use the proportion of zeros in the sampled latents: torch.mean (z==0). This is a simple but informative proxy for energy efficiency in hardware implementations [94–97].

### iP-VAE converges to sparser states while maintaining competitive reconstruction performance.

**Figure 3:**
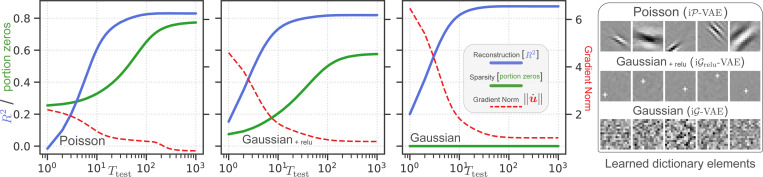
All iterative VAEs converge beyond the training regime $\left(T_{\text{train}}=16\right)$. iP-VAE outperforms $\text{i}G_{\text{relu}}$-VAE in sparsity, while iG-VAE achieves superior reconstruction but with dense representations. iP-VAE maintains reasonable reconstruction performance, despite using constrained representations (sparse, integer-valued spike count). The sparsification dynamics of both iP-VAE and $\text{i}G_{\text{relu}}$-VAE resemble those observed in the mouse visual cortex [98] (appendix C.3). All models are trained on 16 × 16 natural image patches, and the traces are averages over the entire test set. The right panel displays representative dictionary elements (Φ); see Fig. 11 for the complete set of K=512 features.

We frame inference as convergence to attractor states (Fig. 1a) that faithfully represent inputs, achieving high-fidelity reconstruction with sparsity. To evaluate convergence quality, we train iterative VAEs using $T_{\text{train}}=16$, and monitor inference process across $T_{\text{test}}=1,000$ iterations, tracking three metrics averaged over the test set: (1) reconstruction fidelity via $R^{2}$, (2) sparsity via the proportion of zeros, and (3) proximity to the attractor via gradient norm $\left\|\dot{u}\|\propto \|{\text{G}}^{-1}(u)\nabla_{u}F\right\|$ calculated across the latent dimension (K=512). These metrics collectively characterize convergence dynamics and allow us to evaluate attractor quality (Fig. 3).

We determine convergence by detecting when the $R^{2}$ trace flattens and remains stable (details in appendix C.2). For iP-VAE, $\text{i}G_{\text{relu}}$-VAE, and iG-VAE, respectively, we observe convergence times of 95, 75, and 69 iterations. At the final state (t=1,000), these models achieve $R^{2}$ values of 0.83, 0.82, and 0.87, with latents exhibiting 77%, 58%, and 0% zeros (Fig. 3). iP-VAE achieves superior sparsity compared to both Gaussian models, while iG-VAE achieves the best overall $R^{2}$. Throughout inference, the gradient norm $\left\|\dot{u}\right\|$ steadily decreases but plateaus around 0.5, 0.9, and 1.0, respectively, likely due to finite gradient variance induced by stochastic inference dynamics.

### iP-VAE learns V1-like features.

Analysis of the learned features reveals that iP-VAE develops V1-like Gabor filters [99–102], while $\text{i}G_{\text{relu}}$-VAE learns localized pixel-like patterns and iG-VAE learns unstructured features (see right panel in Fig. 3, and Fig. 11 for complete dictionaries). Additionally, when tested with drifting gratings of varying contrasts, iP-VAE exhibits contrast-dependent response latency characteristic of V1 neurons [103, 104] (Fig. 8), possibly a consequence of the normalization dynamics that emerge from our theoretical derivations (eq. (9)). We explore these cortex-like properties further in appendix C.3. In summary, these results suggest iP-VAE learns brain-like representations while achieving the best reconstruction-sparsity compromise.

### iP-VAE achieves the best overall reconstruction-sparsity trade-off.

To assess the robustness of iP-VAE’s performance across hyperparameter settings, we systematically explore combinations of $T_{\text{train}}\in [8,16,32]$, and β values proportional to training iterations (ranging from 0.5× to $4.0\times T_{\text{train}}$). To compare across inference methods, we include amortized counterparts with identical β selection criteria but using $T_{\text{train}}=1$. Finally, we also include LCA [39] due to its theoretical similarity to iP-VAE (eq. (7)). See appendices C.1.1 to C.1.4 for architectural and training details for all models.

**Figure 4:**
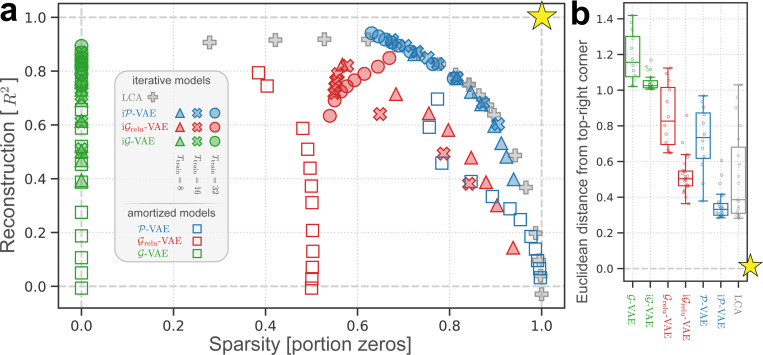
Reconstruction-sparsity trade-off across model families. **(a)** Performance landscape showing reconstruction quality $\left(R^{2}\right)$ versus sparsity (proportion of zeros). Different symbols indicate model variants (triangles: $T_{\text{train}}=8$, crosses: $T_{\text{train}}=16$, circles: $T_{\text{train}}=32$, empty squares: amortized VAEs, plus: LCA), with colors denoting model architectures. The gold star marks the theoretical optimum. **(b)** Overall performance measured as Euclidean distance from the optimum point. Iterative models (right side) consistently outperform their amortized counterparts (left side), with iP-VAE and LCA achieving the best overall performance. See also related Figs. 9 and 10.

Figure 4a positions all models within a unified sparsity-reconstruction landscape, enabling direct comparison of performance metrics and revealing hyperparameter sensitivity. This representation can be interpreted through the lens of rate-distortion theory [69], where $R^{2}$ corresponds to inverse distortion and sparsity to inverse coding rate. The optimal point represents perfect reconstruction $\left(R^{2}=1.0\right)$ using only zeros (sparsity = 1.0). This unachievable ideal is marked with a gold star.

For iP-VAE, varying $T_{\text{train}}$ and β traces a characteristic curve where increased sparsity trades off against reconstruction quality at $T_{\text{train}}=8$, with this trade-off improving at higher training iterations $\left(T_{\text{train}}=16,32\right)$. LCA exhibits a similar pattern, while most other models behave differently. $\text{i}G_{\text{relu}}$-VAE follows this trend at $T_{\text{train}}=8$ but consistently underperforms iP-VAE in both metrics, confirming our observations from Fig. 3. iG-VAE achieves comparable $R^{2}$ values to high-$T_{\text{train}}$ iP-VAE fits but with zero sparsity. These extensive evaluations confirm that both iP-VAE and LCA achieve the best overall reconstruction-sparsity trade-offs among the tested models.

Finally, for fair comparison with LCA’s *maximum a posteriori* (MAP) estimation, we evaluated VAE models with deterministic decoding, where iP-VAE slightly outperforms LCA (Fig. 9). Analysis of β effects in Fig. 10 confirms that higher values increase sparsity in iP-VAE, corroborating theoretical predictions [50]. See appendix C.4 for more details.

### Iterative VAEs unanimously outperform their amortized counterparts.

To quantify overall performance as a single metric, we compute the Euclidean distance from each model to the optimal point (the gold star at $R^{2}=1.0$, sparsity = 1.0), with lower distances indicating better performance. Figure 4b shows that iterative VAE models (iG-VAE, $\text{i}G_{\text{relu}}$-VAE, iP-VAE) consistently outperform their amortized counterparts (G-VAE, $G_{\text{relu}}$-VAE, and P-VAE), despite the latter employing deep convolutional encoders with orders of magnitude more parameters. As predicted by theory (comparing eq. (7) with the LCA dynamics [39]), the performance of LCA and iP-VAE are statistically indistinguishable, though iP-VAE exhibits lower variability across hyperparameter settings.

For clarity in the reconstruction-sparsity analysis, we omit PC and iPC as their dense Gaussian latents are effectively represented by iG-VAE. Extended experiments on MNIST (appendix C.5) show that iterative VAEs outperform PCNs in reconstruction metrics, with iP-VAE achieving the best reconstruction-sparsity trade-off (Table 2). Interestingly, in downstream classification, P-VAE reaches ∼ 98% accuracy, comparable to supervised PCNs [105]. See appendix C.5 for more details.

### iP-VAE exhibits strong out-of-distribution (OOD) generalization.

While the main results use linear decoders $\left(\hat{x}=\text{Φ}z\right)$, our framework easily extends to deep, nonlinear decoders. In appendix B.6, we develop the theory; and in appendix C.6, we train nonlinear iP-VAE models with multilayer perceptron and convolutional decoders—interpretable as *deep sparse coding*—and compare them to hybrid iterative-amortized VAEs [83, 84].

For both within-dataset perturbations (Fig. 13) and cross-dataset settings (Figs. 12, 14 and 15), iP-VAE consistently outperforms alternatives in OOD reconstruction and classification accuracy (Fig. 13). These results suggest that iP-VAE learns a compositional code (Fig. 16), a hypothesis we plan to explore further in future work going beyond reconstruction tasks.

## 5 Conclusions and Discussion

In this paper, we introduced FOND, a framework for deriving brain-like inference dynamics from variational principles. We then applied FOND to derive a new family of iterative VAE models, including the iP-VAE, a spiking model that performs inference in its membrane potential dynamics. iP-VAE’s success likely stems from three key attributes: (1) exponential nonlinearities and (2) emergent normalization, similar to recent advances in sequence modeling [106]; and (3) effective stochastic depth through temporal unrolling, which also explains the effectiveness of hierarchical VAEs [107]. Further, iP-VAE’s weight reuse and its sparse, integer-valued spike count representations enable efficient hardware implementation [108]. While we focused on variational inference, equally important sampling-based approaches offer complementary perspectives [109–114]. Future work should explore acceleration techniques for iterative inference [106], biologically plausible learning rules [115], predictive dynamics for non-stationary sequences [116], hierarchical extensions [117, 118], and neural data applications [119, 120]. We extend upon these points in appendices D.1 to D.5.

In sum, this work connects the prescriptive model development philosophy of FOND with practical algorithms that exhibit brain-like behavior and deliver empirical benefits in machine learning.

## 6 Code and data

Our code, data, and model checkpoints are available here: https://github.com/hadivafaii/IterativeVAE.

## Supplementary Material

Supplement 1

## References

[1] Alhazen. Book of optics (Kitab Al-Manazir). 1011–1021 AD. URL: https://archive.org/details/TheOpticsOfIbnAlHaythamBooksI.

[2] Von Helmholtz Hermann. Handbuch der physiologischen Optik. Vol. 9. Voss, 1867. URL: https://archive.org/details/handbuchderphysi00helm.

[3] Craik Kenneth J. W.. The Nature of Explanation. Cambridge, UK: Cambridge University Press, 1943. URL: https://archive.org/details/natureofexplanat0000crai/page/n5/mode/2up.

[4] Dayan Peter “The Helmholtz machine”. In: Neural Computation 7.5 (1995), pp. 889–904. DOI: 10.1162/neco.1995.7.5.889. 7584891

[5] Lee Tai Singand Mumford David. “Hierarchical Bayesian inference in the visual cortex”. In: JOSA A 20.7 (2003), pp. 1434–1448. DOI: 10.1364/JOSAA.20.001434. 12868647

[6] Barlow Horace. “Redundancy reduction revisited”. In: Network: computation in neural systems 12.3 (2001), p. 241. DOI: 10.1080/net.12.3.241.253. 11563528

[7] Olshausen Bruno A.. “Perception as an Inference Problem”. In: The Cognitive Neurosciences (5th edition) (2014). Ed. by Gazzaniga Michael and Mangun George R.. DOI: 10.7551/mitpress/9504.003.0037. URL: http://rctn.org/bruno/papers/perception-as-inference.pdf.

[8] Ha David and Schmidhuber Jürgen. “World models”. In: (2018). arXiv: 1803.10122v4 [cs.LG].

[9] Hafner Danijar Action and Perception as Divergence Minimization. 2022. arXiv: 2009. 01791 [cs.AI].

[10] Bayes Thomas. “An essay towards solving a problem in the doctrine of chances”. In: Philosophical Transactions of the Royal Society of London 53 (1763), pp. 370–418. DOI: 10.1098/rstl.1763.0053.

[11] Laplace Pierre-Simon. Théorie analytique des probabilités. Courcier, 1812. URL: https://www.probabilityandfinance.com/pulskamp/Laplace/TAP_final-book_I.pdf.

[12] Murphy Kevin P. Machine Learning: A Probabilistic Perspective. MIT Press, 2012. URL: https://probml.github.io/pml-book/book0.html.

[13] Neal Radford M. and Hinton Geoffrey E.. “A View of the Em Algorithm that Justifies Incremental, Sparse, and other Variants”. In: Learning in Graphical Models. Ed. by Jordan Michael I.. Dordrecht: Springer Netherlands, 1998, pp. 355–368. ISBN: 978–94-011–5014-9. DOI: 10.1007/978-94-011-5014-9_12.

[14] Zellner Arnold and. “Optimal Information Processing and Bayes’s Theorem”. In: The American Statistician 42.4 (1988), pp. 278–280. DOI: 10.1080/00031305.1988.10475585.

[15] Jordan Michael I “An introduction to variational methods for graphical models”. In: Machine learning 37 (1999), pp. 183–233. DOI: 10.1023/A:1007665907178.

[16] Tzikas Dimitris G “The variational approximation for Bayesian inference”. In: IEEE Signal Processing Magazine 25.6 (2008), pp. 131–146. DOI: 10.1109/MSP.2008.929620.

[17] Blei David M. “Variational Inference: A Review for Statisticians”. In: Journal of the American Statistical Association 112.518 (2017), pp. 859–877. DOI: 10.1080/01621459.2017.1285773.

[18] Kingma Diederik Pand Welling Max. “Auto-encoding variational bayes”. In: (2014). arXiv: 1312.6114v11 [stat.ML].

[19] Rezende Danilo Jimenez “Stochastic backpropagation and approximate inference in deep generative models”. In: International Conference on Machine Learning. PMLR. 2014, pp. 1278–1286. URL: https://proceedings.mlr.press/v32/rezende14.html.

[20] Kingma Diederik Pand Welling Max. “An introduction to variational autoencoders”. In: Foundations and Trends^®^ in Machine Learning 12.4 (2019), pp. 307–392. DOI: 10.1561/2200000056.

[21] Marino Joseph. “Predictive coding, variational autoencoders, and biological connections”. In: Neural Computation 34.1 (2022), pp. 1–44. DOI: 10.1162/neco_a_01458. 34758480

[22] Rao Rajesh PNand Ballard Dana H. “Predictive coding in the visual cortex: a functional interpretation of some extra-classical receptive-field effects”. In: Nature Neuroscience 2.1 (1999), pp. 79–87. DOI: 10.1038/4580. 10195184

[23] Friston Karl. “A theory of cortical responses”. In: Philosophical transactions of the Royal Society B: Biological Sciences 360.1456 (2005), pp. 815–836. DOI: 10.1098/rstb.2005.1622. PMC1569488 15937014

[24] Friston Karl. “The free-energy principle: a unified brain theory?” In: Nature Reviews Neuroscience 11.2 (2010), pp. 127–138. DOI: 10.1038/nrn2787. 20068583

[25] Bogacz Rafal. “A tutorial on the free-energy framework for modelling perception and learning”. In: Journal of Mathematical Psychology 76 (2017). Model-based Cognitive Neuroscience, pp. 198–211. ISSN: 0022–2496. DOI: 10.1016/j.jmp.2015.11.003. PMC5341759 28298703

[26] Millidge Beren “Predictive Coding: Towards a Future of Deep Learning beyond Backpropagation?” In: International Joint Conference on Artificial Intelligence. 2022. DOI: 10.24963/ijcai.2022/774.

[27] Gershman Samuel J. “What does the free energy principle tell us about the brain?” In: arXiv:1901.07945 (2019). URL: https://arxiv.org/abs/1901.07945.

[28] Andrews Mel. “The math is not the territory: navigating the free energy principle”. In: Biology & Philosophy 36.3 (2021), p. 30. DOI: 10.1007/s10539-021-09807-0.

[29] Sohl-Dickstein Jascha “Deep unsupervised learning using nonequilibrium thermodynamics”. In: International conference on machine learning. PMLR. 2015, pp. 2256–2265.

[30] Kingma Diederik Pand Gao Ruiqi. “Understanding Diffusion Objectives as the ELBO with Simple Data Augmentation”. In: Thirty-seventh Conference on Neural Information Processing Systems. 2023. URL: https://openreview.net/forum?id=NnMEadcdyD.

[31] Luo Calvin. Understanding Diffusion Models: A Unified Perspective. 2022. arXiv: 2208. 11970 [cs.LG].

[32] Srinivasan Mandyam Veerambudi “Predictive coding: a fresh view of inhibition in the retina”. In: Proceedings of the Royal Society of London. Series B. Biological Sciences 216.1205 (1982), pp. 427–459. DOI: 10.1098/rspb.1982.0085. 6129637

[33] Millidge Beren Predictive Coding: a Theoretical and Experimental Review. 2022. arXiv: 2107.12979 [cs.AI].

[34] Khan Mohammad Emtiyazand Rue HÃ¥vard. “The Bayesian Learning Rule”. In: Journal of Machine Learning Research 24.281 (2023), pp. 1–46. URL: http://jmlr.org/papers/v24/22-0291.html.

[35] Amari Shun-ichi. “Natural Gradient Works Efficiently in Learning”. In: Neural Computation 10.2 (Feb. 1998), pp. 251–276. ISSN: 0899–7667. DOI: 10.1162/089976698300017746.

[36] Gershman Samuel and Goodman Noah. “Amortized inference in probabilistic reasoning”. In: Proceedings of the annual meeting of the cognitive science society. Vol. 36. 36. 2014. URL: https://escholarship.org/uc/item/34j1h7k5.

[37] Amos Brandon. “Tutorial on Amortized Optimization”. In: Foundations and Trends^®^ in Machine Learning 16.5 (2023), pp. 592–732. ISSN: 1935–8237. DOI: 10.1561/2200000102.

[38] Ganguly Ankush “Amortized Variational Inference: A Systematic Review”. In: Journal of Artificial Intelligence Research 78 (2023), pp. 167–215. DOI: 10.1613/jair.1.14258.

[39] Rozell Christopher J “Sparse coding via thresholding and local competition in neural circuits”. In: Neural Computation 20.10 (2008), pp. 2526–2563. DOI: 10.1162/neco.2008.03-07-486. 18439138

[40] Lamme Victor A.F. and Roelfsema Pieter R.. “The distinct modes of vision offered by feedforward and recurrent processing”. In: Trends in Neurosciences 23.11 (2000), pp. 571–579. ISSN: 0166–2236. DOI: 10.1016/S0166-2236(00)01657-X. 11074267

[41] Kietzmann Tim C “Recurrence is required to capture the representational dynamics of the human visual system”. In: Proceedings of the National Academy of Sciences 116.43 (2019), pp. 21854–21863. DOI: 10.1073/pnas.1905544116. PMC6815174 31591217

[42] Kar Kohitij “Evidence that recurrent circuits are critical to the ventral stream’s execution of core object recognition behavior”. In: Nature neuroscience 22.6 (2019), pp. 974–983. DOI: 10.1038/s41593-019-0392-5. 31036945 PMC8785116

[43] van Bergen Ruben Sand Kriegeskorte Nikolaus. “Going in circles is the way forward: the role of recurrence in visual inference”. In: Current Opinion in Neurobiology 65 (2020). Whole-brain interactions between neural circuits, pp. 176–193. ISSN: 0959–4388. DOI: 10.1016/j.conb.2020.11.009. 33279795

[44] Shi Yuelin “Rapid, concerted switching of the neural code in inferotemporal cortex”. In: bioRxiv (2023). DOI: 10.1101/2023.12.06.570341. PMC13148990 41882367

[45] Kohn Adam. “Visual adaptation: physiology, mechanisms, and functional benefits”. In: Journal of neurophysiology 97.5 (2007), pp. 3155–3164. DOI: 10.1152/jn.00086.2007. 17344377

[46] Fischer Jason and Whitney David. “Serial dependence in visual perception”. In: Nature neuroscience 17.5 (2014), pp. 738–743. DOI: 10.1038/nn.3689. 24686785 PMC4012025

[47] Cicchini Guido Marco “Serial dependence in perception”. In: Annual Review of Psychology 75 (2024), pp. 129–154. DOI: 10.1146/annurev-psych-021523-104939. 37758238

[48] Freyd Jennifer Jand Finke Ronald A. “Representational momentum”. In: Journal of Experimental Psychology: Learning, Memory, and Cognition 10.1 (1984), p. 126. DOI: 10.1037/0278-7393.10.1.126.

[49] De Lange Floris P “How do expectations shape perception?” In: Trends in cognitive sciences 22.9 (2018), pp. 764–779. DOI: 10.1016/j.tics.2018.06.002. 30122170

[50] Vafaii Hadi “Poisson Variational Autoencoder”. In: The Thirty-eighth Annual Conference on Neural Information Processing Systems. 2024. URL: https://openreview.net/forum?id=ektPEcqGLb.

[51] Tolhurst David J “The statistical reliability of signals in single neurons in cat and monkey visual cortex”. In: Vision research 23.8 (1983), pp. 775–785. DOI: 10.1016/0042-6989(83)90200-6. 6623937

[52] Dean AF. “The variability of discharge of simple cells in the cat striate cortex”. In: Experimental Brain Research 44.4 (1981), pp. 437–440. DOI: 10.1007/BF00238837. 7308358

[53] Pouget Alexandre “Information processing with population codes”. In: Nature Reviews Neuroscience 1.2 (2000), pp. 125–132. DOI: 10.1038/35039062. 11252775

[54] Olshausen Bruno Aand Field David J. “Emergence of simple-cell receptive field properties by learning a sparse code for natural images”. In: Nature 381.6583 (1996), pp. 607–609. DOI: 10.1038/381607a0. 8637596

[55] Olshausen Bruno A. “Learning linear, sparse, factorial codes”. In: (1996). URL: https://dspace.mit.edu/bitstream/handle/1721.1/7184/AIM-1580.pdf.

[56] Dayan Peter and Abbott Laurence F. “Theoretical Neuroscience”. In: (2001). URL: https://mitpress.mit.edu/9780262041997/theoretical-neuroscience/.

[57] Khan Mohammad Emtiyazand Nielsen Didrik. “Fast yet simple natural-gradient descent for variational inference in complex models”. In: 2018 International Symposium on Information Theory and Its Applications (ISITA). IEEE. 2018, pp. 31–35. DOI: 10.23919/ISITA.2018.8664326.

[58] Catoni Olivier. PAC-Bayesian Supervised Classification: The Thermodynamics of Statistical Learning. Institute of Mathematical Statistics Lecture Notes—Monograph Series. IMS, 2007. DOI: 10.1214/074921707000000391. URL: https://arxiv.org/abs/0712.0248.

[59] Bissiri P. G. “A general framework for updating belief distributions”. In: Journal of the Royal Statistical Society: Series B (Statistical Methodology) 78.5 (2016), pp. 1103–1130. DOI: 10.1111/rssb.12158. PMC5082587 27840585

[60] Jones Matt “Bayesian Online Natural Gradient (BONG)”. In: The Thirty-eighth Annual Conference on Neural Information Processing Systems. 2024. URL: https://openreview.net/forum?id=E7en5DyO2G.

[61] Rybkin Oleh “Simple and Effective VAE Training with Calibrated Decoders”. In: Proceedings of the 38th International Conference on Machine Learning. Ed. by Meila Marinaand Zhang Tong. Vol. 139. Proceedings of Machine Learning Research. PMLR, June 2021, pp. 9179–9189. URL: https://proceedings.mlr.press/v139/rybkin21a.html.

[62] Priebe Nicholas J “The contribution of spike threshold to the dichotomy of cortical simple and complex cells”. In: Nature neuroscience 7.10 (2004), pp. 1113–1122. DOI: 10.1038/nn1310. 15338009 PMC2915829

[63] Fourcaud-Trocmé Nicolas “How spike generation mechanisms determine the neuronal response to fluctuating inputs”. In: Journal of neuroscience 23.37 (2003), pp. 11628–11640. DOI: 10.1523/JNEUROSCI.23-37-11628.2003. 14684865 PMC6740955

[64] Alfred North Whitehead. Process and Reality: An Essay in Cosmology. 1929. URL: https://archive.org/details/processrealitygi00alfr.

[65] J J. “Neural networks and physical systems with emergent collective computational abilities.” In: Proceedings of the National Academy of Sciences 79.8 (1982), pp. 2554–2558. DOI: 10.1073/pnas.79.8.2554. PMC346238 6953413

[66] Friston Karl “Active Inference: A Process Theory”. In: Neural Computation 29.1 (Jan. 2017), pp. 1–49. ISSN: 0899–7667. DOI: 10.1162/NECO_a_00912. 27870614

[67] Vyas Saurabh “Computation Through Neural Population Dynamics”. In: Annual Review of Neuroscience 43.Volume 43, 2020 (2020), pp. 249–275. ISSN: 1545–4126. DOI: 10.1146/annurev-neuro-092619-094115. PMC7402639 32640928

[68] Amari Shun-ichi and Nagaoka Hiroshi. Methods of information geometry. Vol. 191. American Mathematical Soc., 2000. DOI: 10.1090/mmono/191.

[69] Alemi Alexander “Fixing a Broken ELBO”. In: Proceedings of the 35th International Conference on Machine Learning. Ed. by Dy Jenniferand Krause Andreas. Vol. 80. Proceedings of Machine Learning Research. PMLR, July 2018, pp. 159–168. URL: https://proceedings.mlr.press/v80/alemi18a.html.

[70] Higgins Irina “beta-VAE: Learning Basic Visual Concepts with a Constrained Variational Framework”. In: International Conference on Learning Representations. 2017. URL: https://openreview.net/forum?id=Sy2fzU9gl.

[71] Bengio Yoshua “Estimating or Propagating Gradients Through Stochastic Neurons for Conditional Computation”. In: (2013). arXiv: 1308.3432 [cs.LG].

[72] Amari Shun-Ichi. “Characteristics of random nets of analog neuron-like elements”. In: IEEE Transactions on systems, man, and cybernetics 5 (1972), pp. 643–657. DOI: 10.1109/TSMC. 1972.4309193.

[73] Gerstner Wulfram and Kistler Werner M.. Spiking Neuron Models: Single Neurons, Populations, Plasticity. Cambridge, UK: Cambridge University Press, 2002. ISBN: 9780521890793. DOI: 10.1017/CBO9780511815706.

[74] Gerstner Wulfram Neuronal dynamics: From single neurons to networks and models of cognition. Cambridge University Press, 2014. DOI: 10.1017/CBO9781107447615.

[75] Rubin Daniel B “The stabilized supralinear network: a unifying circuit motif underlying multi-input integration in sensory cortex”. In: Neuron 85.2 (2015), pp. 402–417. DOI: 10.1016/j.neuron.2014.12.026. PMC4344127 25611511

[76] Kandel Eric R. Principles of Neural Science. 5th ed. New York: McGraw Hill, 2000. URL: https://archive.org/details/PrinciplesOfNeuralScienceFifthKANDEL.

[77] Ko Ho “The emergence of functional microcircuits in visual cortex”. In: Nature 496.7443 (2013), pp. 96–100. DOI: 10.1038/nature12015. PMC4843961 23552948

[78] Chettih Selmaan Nand Harvey Christopher D. “Single-neuron perturbations reveal feature-specific competition in V1”. In: Nature 567.7748 (2019), pp. 334–340. DOI: 10.1038/s41586-019-0997-6. PMC6682407 30842660

[79] Ding Zhuokun “Functional connectomics reveals general wiring rule in mouse visual cortex”. In: Nature 640.8058 (2025), pp. 459–469. DOI: 10.1038/s41586-025-08840-3. PMC11981947 40205211

[80] Gregor Karol and LeCun Yann. “Learning fast approximations of sparse coding”. In: Proceedings of the 27th international conference on international conference on machine learning. 2010, pp. 399–406. URL: https://icml.cc/Conferences/2010/papers/449.pdf.

[81] Whittington James C. R. “Disentanglement with Biological Constraints: A Theory of Functional Cell Types”. In: The Eleventh International Conference on Learning Representations. 2023. URL: https://openreview.net/forum?id=9Z_GfhZnGH.

[82] Salvatori Tommaso “A Stable, Fast, and Fully Automatic Learning Algorithm for Predictive Coding Networks”. In: The Twelfth International Conference on Learning Representations. 2024. URL: https://openreview.net/forum?id=RyUvzda8GH.

[83] Marino Joe “Iterative Amortized Inference”. In: Proceedings of the 35th International Conference on Machine Learning. Ed. by Dy Jenniferand Krause Andreas. Vol. 80. Proceedings of Machine Learning Research. PMLR, July 2018, pp. 3403–3412. URL: https://proceedings.mlr.press/v80/marino18a.html.

[84] Kim Yoon “Semi-Amortized Variational Autoencoders”. In: Proceedings of the 35th International Conference on Machine Learning. Ed. by Dy Jenniferand Krause Andreas. Vol. 80. Proceedings of Machine Learning Research. PMLR, July 2018, pp. 2678–2687. URL: https://proceedings.mlr.press/v80/kim18e.html.

[85] Werbos Paul J. “Backpropagation through time: what it does and how to do it”. In: Proceedings of the IEEE 78.10 (1990), pp. 1550–1560. DOI: 10.1109/5.58337.

[86] Lillicrap Timothy Pand Santoro Adam. “Backpropagation through time and the brain”. In: Current Opinion in Neurobiology 55 (2019). Machine Learning, Big Data, and Neuroscience, pp. 82–89. ISSN: 0959–4388. DOI: 10.1016/j.conb.2019.01.011. URL: https://www.sciencedirect.com/science/article/pii/S0959438818302009. 30851654

[87] Jastrzębski Stanisław Residual Connections Encourage Iterative Inference. 2018. arXiv: 1710.04773 [cs.CV].

[88] Liao Qianli and Poggio Tomaso. Bridging the Gaps Between Residual Learning, Recurrent Neural Networks and Visual Cortex. 2020. arXiv: 1604.03640 [cs.LG].

[89] Schwarzschild Avi. “Deep Thinking Systems: Logical Extrapolation With Recurrent Neural Networks”. PhD thesis. UMD, 2023, p. 124. URL: https://www.proquest.com/dissertations-theses/deep-thinking-systems-logical-extrapolation-with/docview/2830027656/se-2.

[90] Zhou Kaiyang “Domain generalization: A survey”. In: IEEE Transactions on Pattern Analysis and Machine Intelligence 45.4 (2022), pp. 4396–4415. DOI: 10.1109/TPAMI.2022.3195549. 35914036

[91] Wang Jindong “Generalizing to unseen domains: A survey on domain generalization”. In: IEEE transactions on knowledge and data engineering 35.8 (2022), pp. 8052–8072. DOI: 10.1109/TKDE.2022.3178128.

[92] van Hateren J. H. and van der Schaaf A.. “Independent component filters of natural images compared with simple cells in primary visual cortex”. In: Proceedings of the Royal Society of London. Series B: Biological Sciences 265.1394 (1998), pp. 359–366. DOI: 10.1098/rspb.1998.0303. PMC1688904 9523437

[93] LeCun Yann “Gradient-based learning applied to document recognition”. In: Proceedings of the IEEE 86.11 (1998), pp. 2278–2324. DOI: 10.1109/5.726791.

[94] Han Song “EIE: Efficient inference engine on compressed deep neural network”. In: ACM SIGARCH Computer Architecture News 44.3 (2016), pp. 243–254. DOI: 10.1145/3007787.3001163.

[95] Sze Vivienne Efficient processing of deep neural networks. Springer, 2020. DOI: 10.1007/978-3-031-01766-7.

[96] Gholami Amir “A survey of quantization methods for efficient neural network inference”. In: Low-power computer vision. Chapman and Hall/CRC, 2022, pp. 291–326. DOI: 10.1201/9781003162810-13.

[97] Hubara Itay “Binarized Neural Networks”. In: Advances in Neural Information Processing Systems. Ed. by Lee D.et al. Vol. 29. Curran Associates, Inc., 2016. URL: https://proceedings.neurips.cc/paper_files/paper/2016/hash/d8330f857a17c53d217014ee776bfd50-Abstract.html.

[98] Amin Moosavi S. “Temporal dynamics of energy-efficient coding in mouse primary visual cortex”. In: bioRxiv (2024). DOI: 10.1101/2024.12.17.628997.

[99] Marĉelja S.. “Mathematical description of the responses of simple cortical cells*”. In: J. Opt. Soc. Am. 70.11 (Nov. 1980), pp. 1297–1300. DOI: 10.1364/JOSA.70.001297. 7463179

[100] Hubel David H. and Wiesel Torsten N.. “Receptive fields of single neurones in the cat’s striate cortex”. In: The Journal of Physiology 148 (1959). DOI: 10.1113/jphysiol.1959.sp006308. PMC1363130 14403679

[101] Hubel David H. and Wiesel Torsten N.. “Receptive fields and functional architecture of monkey striate cortex”. In: The Journal of Physiology 195 (1968). DOI: 10.1113/jphysiol.1968.sp008455. PMC1557912 4966457

[102] Fu Jiakun “Heterogeneous orientation tuning in the primary visual cortex of mice diverges from Gabor-like receptive fields in primates”. In: Cell reports 43.8 (2024). DOI: 10.1016/j.celrep.2024.114639. PMC11463840 39167488

[103] Carandini Matteo “Linearity and normalization in simple cells of the macaque primary visual cortex”. In: Journal of Neuroscience 17.21 (1997), pp. 8621–8644. DOI: 10.1523/JNEUROSCI.17-21-08621.1997. 9334433 PMC6573724

[104] Albrecht Duane G “Visual cortex neurons of monkeys and cats: temporal dynamics of the contrast response function”. In: Journal of neurophysiology 88.2 (2002), pp. 888–913. DOI: 10.1152/jn.2002.88.2.888. 12163540

[105] Pinchetti Luca “Benchmarking Predictive Coding Networks – Made Simple”. In: The Thirteenth International Conference on Learning Representations. 2025. URL: https://openreview.net/forum?id=sahQq2sH5x.

[106] Beck Maximilian “xLSTM: Extended Long Short-Term Memory”. In: The Thirty-eighth Annual Conference on Neural Information Processing Systems. 2024. URL: https://openreview.net/forum?id=ARAxPPIAhq.

[107] Child Rewon. “Very Deep {VAE}s Generalize Autoregressive Models and Can Outperform Them on Images”. In: International Conference on Learning Representations. 2021. URL: https://openreview.net/forum?id=RLRXCV6DbEJ.

[108] Schuman C. D. “Opportunities for neuromorphic computing algorithms and applications”. In: Nature Computational Science (2022). DOI: 10.1038/s43588-022-00223-2. 38177712

[109] Hoyer Patrik and Hyvärinen Aapo. “Interpreting Neural Response Variability as Monte Carlo Sampling of the Posterior”. In: Advances in Neural Information Processing Systems. Ed. By Becker S. Vol. 15. MIT Press, 2002. URL: https://proceedings.neurips.cc/paper_files/paper/2002/hash/a486cd07e4ac3d270571622f4f316ec5-Abstract.html.

[110] Orbán Gergő “Neural variability and sampling-based probabilistic representations in the visual cortex”. In: Neuron 92.2 (2016), pp. 530–543. DOI: 10.1016/j.neuron.2016.09.038. PMC5077700 27764674

[111] Masset Paul “Natural gradient enables fast sampling in spiking neural networks”. In: Advances in Neural Information Processing Systems. Ed. by Koyejo S.et al. Vol. 35. Curran Associates, Inc., 2022, pp. 22018–22034. URL: https://proceedings.neurips.cc/paper_files/paper/2022/hash/8a0fd48510590071e3c129a79b8b8527-Abstract-Conference.html. PMC10358281 37476623

[112] Fang Michael Y.-S. “Learning and Inference in Sparse Coding Models With Langevin Dynamics”. In: Neural Computation 34.8 (July 2022), pp. 1676–1700. ISSN: 0899–7667. DOI: 10.1162/neco_a_01505. 35798329

[113] Oliviers Gaspard “Learning probability distributions of sensory inputs with Monte Carlo predictive coding”. In: PLOS Computational Biology 20.10 (Oct. 2024), pp. 1–34. DOI: 10.1371/journal.pcbi.1012532. PMC11524488 39475902

[114] Sennesh Eli Zachary “Divide-and-Conquer Predictive Coding: a structured Bayesian inference algorithm”. In: The Thirty-eighth Annual Conference on Neural Information Processing Systems. 2024. URL: https://openreview.net/forum?id=dxwIaCVkWU.

[115] Zylberberg Joel “A Sparse Coding Model with Synaptically Local Plasticity and Spiking Neurons Can Account for the Diverse Shapes of V1 Simple Cell Receptive Fields”. In: PLOS Computational Biology 7.10 (Oct. 2011), pp. 1–12. DOI: 10.1371/journal.pcbi.1002250. PMC3203062 22046123

[116] Duran-Martin Gerardo “A unifying framework for generalised Bayesian online learning in non-stationary environments”. In: Transactions on Machine Learning Research (2025). ISSN: 2835–8856. URL: https://openreview.net/forum?id=osesw2V10u.

[117] Vafaii Hadi “Hierarchical VAEs provide a normative account of motion processing in the primate brain”. In: Thirty-seventh Conference on Neural Information Processing Systems. 2023. URL: https://openreview.net/forum?id=1wOkHN9JK8.

[118] Csikor Ferenc “Top-down perceptual inference shaping the activity of early visual cortex”. In: bioRxiv (2025). DOI: 10.1101/2023.11.29.569262. PMC12618887 41238593

[119] Schrimpf Martin “Brain-score: Which artificial neural network for object recognition is most brain-like?” In: BioRxiv (2018), p. 407007. DOI: 10.1101/407007.

[120] Wang Eric Y “Foundation model of neural activity predicts response to new stimulus types”. In: Nature 640.8058 (2025), pp. 470–477. DOI: 10.1038/s41586-025-08829-y. PMC11981942 40205215

[121] Paszke Adam “PyTorch: An Imperative Style, High-Performance Deep Learning Library”. In: Advances in Neural Information Processing Systems. Vol. 32. Curran Associates, Inc., 2019. URL: https://papers.nips.cc/paper_files/paper/2019/hash/bdbca288fee7f92f2bfa9f7012727740-Abstract.html.

[122] Harris Charles R. “Array programming with NumPy”. In: Nature 585.7825 (Sept. 2020), pp. 357–362. DOI: 10.1038/s41586-020-2649-2. PMC7759461 32939066

[123] Virtanen Pauli “SciPy 1.0: Fundamental Algorithms for Scientific Computing in Python”. In: Nature Methods 17 (2020), pp. 261–272. DOI: 10.1038/s41592-019-0686-2. PMC7056644 32015543

[124] Pedregosa Fabian “Scikit-learn: Machine learning in Python”. In: the Journal of machine Learning research 12 (2011), pp. 2825–2830. DOI: 10.5555/1953048.2078195.

[125] The pandas development team. “pandas-dev/pandas: Pandas”. Version latest. In: (Feb. 2020). DOI: 10.5281/zenodo.3509134.

[126] Hunter John D. “Matplotlib: A 2D graphics environment”. In: Computing in science & engineering 9.03 (2007), pp. 90–95. DOI: 10.1109/MCSE.2007.55.

[127] Waskom Michael L. “Seaborn: statistical data visualization”. In: Journal of Open Source Software 6.60 (2021), p. 3021. DOI: 10.21105/joss.03021.

[128] Kant Immanuel. Critique of Pure Reason. Translated by Guyer Pauland Wood Allen W., Cambridge University Press. 1781. DOI: 10.1017/CBO9780511804649.

[129] Aristotle. Posterior Analytics. Trans. by Jonathan Barnes. Originally written c. 350 BCE. Oxford: Oxford University Press, 1994. URL: https://classics.mit.edu/Aristotle/posterior.1.i.html.

[130] Locke John. An essay concerning human understanding. Ed. by Nidditch Peter H.. Oxford University Press, 1690. DOI: 10.1093/actrade/9780198243861.book.1.

[131] Gopnik Alison The Scientist in the Crib: What Early Learning Tells Us About the Mind. HarperCollins, 2004. URL: http://alisongopnik.com/TheScientistInTheCrib.htm.

[132] Plato. Meno. Trans. by Grube G.M.A.. Originally written c. 380 BCE. Indianapolis: Hackett Publishing, 1976. URL: https://classics.mit.edu/Plato/meno.html.

[133] Spelke Elizabeth S. and Kinzler Katherine D.. “Core knowledge”. In: Developmental Science 10.1 (2007). DOI: 10.1111/j.1467-7687.2007.00569.x. 17181705

[134] Anthony M Zador. “A critique of pure learning and what artificial neural networks can learn from animal brains”. In: Nature Communications 10.1 (2019), p. 3770. DOI: 10.1038/s41467-019-11786-6. PMC6704116 31434893

[135] Boring Edwin Garrigues. “Sensation and perception in the history of experimental psychology.” In: (1942). URL: https://ia801507.us.archive.org/12/items/in.ernet.dli.2015.52372/2015.52372.Sensation-And-Perception-In-The-History-Of-Experimental-Psychology_text.pdf.

[136] Gregory Richard Langton. “Perceptions as hypotheses”. In: Philosophical Transactions of the Royal Society of London. B, Biological Sciences 290.1038 (1980), pp. 181–197. DOI: 10.1098/RSTB.1980.0090. 6106237

[137] Clark Andy. “Whatever next? Predictive brains, situated agents, and the future of cognitive science”. In: Behavioral and brain sciences 36.3 (2013), pp. 181–204. DOI: 10.1017/S0140525X12000477. 23663408

[138] Knill David Cand Pouget Alexandre. “The Bayesian brain: the role of uncertainty in neural coding and computation”. In: Trends in Neurosciences 27.12 (2004), pp. 712–719. DOI: 10.1016/j.tins.2004.10.007. 15541511

[139] Friston Karl. “The free-energy principle: a rough guide to the brain?” In: Trends in cognitive sciences 13.7 (2009), pp. 293–301. DOI: 10.1016/j.neuron.2016.03.020. 19559644

[140] Friston Karl “The free energy principle made simpler but not too simple”. In: Physics Reports 1024 (2023). The free energy principle made simpler but not too simple, pp. 1–29. ISSN: 0370–1573. DOI: 10.1016/j.physrep.2023.07.001.

[141] Olshausen Bruno A. and Field David J.. “Sparse coding with an overcomplete basis set: A strategy employed by V1?” In: Vision Research 37.23 (1997), pp. 3311–3325. ISSN: 0042–6989. DOI: 10.1016/S0042-6989(97)00169-7. 9425546

[142] Olshausen Bruno Aand Field David J. “Sparse coding of sensory inputs”. In: Current opinion in neurobiology 14.4 (2004), pp. 481–487. DOI: 10.1016/j.conb.2004.07.007. 15321069

[143] Lotter William “Deep Predictive Coding Networks for Video Prediction and Unsupervised Learning”. In: International Conference on Learning Representations. 2017. URL: https://openreview.net/forum?id=B1ewdt9xe.

[144] Buckley Christopher L. “The free energy principle for action and perception: A mathematical review”. In: Journal of Mathematical Psychology 81 (2017), pp. 55–79. ISSN: 0022–2496. DOI: 10.1016/j.jmp.2017.09.004.

[145] Thomas M Joy A. Thomas. Cover. Elements of Information Theory. 2nd ed. Wiley-Interscience, 2006. DOI: 10.1002/047174882X.

[146] Tishby Naftali The information bottleneck method. 2000. arXiv: physics/0004057 [physics.data-an].

[147] Landau L. D. and Lifshitz E. M.. Statistical Physics, Part 1. 3rd. Oxford: Pergamon Press, 1980. URL: https://ia902908.us.archive.org/31/items/ost-physics-landaulifshitz-statisticalphysics/LandauLifshitz-StatisticalPhysics.pdf.

[148] Jaynes E. T.. Probability Theory: The Logic of Science. Cambridge: Cambridge University Press, 2003. URL: http://www.med.mcgill.ca/epidemiology/hanley/bios601/GaussianModel/JaynesProbabilityTheory.pdf.

[149] Hinton Geoffrey Eand Zemel Richard. “Autoencoders, Minimum Description Length and Helmholtz Free Energy”. In: Advances in Neural Information Processing Systems. Ed. By Cowan J. Vol. 6. Morgan-Kaufmann, 1993. URL: https://proceedings.neurips.cc/paper/1993/hash/9e3cfc48eccf81a0d57663e129aef3cb-Abstract.html.

[150] Dai Bin and Wipf David. Diagnosing and Enhancing VAE Models. 2019. arXiv: 1903.05789 [cs.LG].

[151] Sønderby Casper Kaae “Ladder Variational Autoencoders”. In: Advances in Neural Information Processing Systems. Vol. 29. Curran Associates, Inc., 2016. URL: https://papers.nips.cc/paper_files/paper/2016/hash/6ae07dcb33ec3b7c814df797cbda0f87-Abstract.html.

[152] Vahdat Arash and Kautz Jan. “NVAE: A Deep Hierarchical Variational Autoencoder”. In: Advances in Neural Information Processing Systems. Vol. 33. Curran Associates, Inc., 2020, pp. 19667–19679. URL: https://papers.nips.cc/paper_files/paper/2020/hash/e3b21256183cf7c2c7a66be163579d37-Abstract.html.

[153] Tomczak Jakub and Welling Max. “VAE with a VampPrior”. In: Proceedings of the Twenty-First International Conference on Artificial Intelligence and Statistics. Ed. by Storkey Amosand Perez-Cruz Fernando. Vol. 84. Proceedings of Machine Learning Research. PMLR, Apr. 2018, pp. 1214–1223. URL: https://proceedings.mlr.press/v84/tomczak18a.html.

[154] Nalisnick Eric and Smyth Padhraic. “Stick-Breaking Variational Autoencoders”. In: International Conference on Learning Representations. 2017. URL: https://openreview.net/forum?id=S1jmAotxg.

[155] Loaiza-Ganem Gabrieland Cunningham John P. “The continuous Bernoulli: fixing a pervasive error in variational autoencoders”. In: Advances in Neural Information Processing Systems. Ed. by Wallach H. Vol. 32. Curran Associates, Inc., 2019. URL: https://proceedings.neurips.cc/paper_files/paper/2019/file/f82798ec8909d23e55679ee26bb26437-Paper.pdf.

[156] Pandarinath Chethan “Inferring single-trial neural population dynamics using sequential auto-encoders”. In: Nature methods 15.10 (2018), pp. 805–815. DOI: 10.1038/s41592-018-0109-9. PMC6380887 30224673

[157] Zhao He “Variational Autoencoders for Sparse and Overdispersed Discrete Data”. In: Proceedings of the Twenty Third International Conference on Artificial Intelligence and Statistics. Ed. by Chiappa Silviaand Calandra Roberto. Vol. 108. Proceedings of Machine Learning Research. PMLR, Aug. 2020, pp. 1684–1694. URL: https://proceedings.mlr.press/v108/zhao20c.html.

[158] Salimans Tim “PixelCNN++: Improving the PixelCNN with Discretized Logistic Mixture Likelihood and Other Modifications”. In: International Conference on Learning Representations. 2017. URL: https://openreview.net/forum?id=BJrFC6ceg.

[159] Rezende Danilo and Mohamed Shakir. “Variational Inference with Normalizing Flows”. In: Proceedings of the 32nd International Conference on Machine Learning. Ed. by Bach Francisand Blei David. Vol. 37. Proceedings of Machine Learning Research. Lille, France: PMLR, July 2015, pp. 1530–1538. URL: https://proceedings.mlr.press/v37/rezende15.html.

[160] Kingma Durk P “Improved Variational Inference with Inverse Autoregressive Flow”. In: Advances in Neural Information Processing Systems. Ed. by Lee D.et al. Vol. 29. Curran Associates, Inc., 2016. URL: https://proceedings.neurips.cc/paper_files/paper/2016/hash/ddeebdeefdb7e7e7a697e1c3e3d8ef54-Abstract.html.

[161] Jang Eric “Categorical Reparameterization with Gumbel-Softmax”. In: International Conference on Learning Representations. 2017. URL: https://openreview.net/forum?id=rkE3y85ee.

[162] Maddison Chris J. “The Concrete Distribution: A Continuous Relaxation of Discrete Random Variables”. In: International Conference on Learning Representations. 2017. URL: https://openreview.net/forum?id=S1jE5L5gl.

[163] Rolfe Jason Tyler. “Discrete Variational Autoencoders”. In: International Conference on Learning Representations. 2017. URL: https://openreview.net/forum?id=ryMxXPFex.

[164] Vahdat Arash “DVAE++: Discrete Variational Autoencoders with Overlapping Transformations”. In: Proceedings of the 35th International Conference on Machine Learning. Ed. by Dy Jenniferand Krause Andreas. Vol. 80. Proceedings of Machine Learning Research. PMLR, July 2018, pp. 5035–5044. URL: https://proceedings.mlr.press/v80/vahdat18a.html.

[165] Park Yookoon “Variational Laplace Autoencoders”. In: Proceedings of the 36th International Conference on Machine Learning. Ed. by Kamalika Chaudhuri and Ruslan Salakhutdinov. Vol. 97. Proceedings of Machine Learning Research. PMLR, June 2019, pp. 5032–5041. URL: https://proceedings.mlr.press/v97/park19a.html.

[166] Srivastava Akash and Sutton Charles. “Autoencoding Variational Inference For Topic Models”. In: International Conference on Learning Representations. 2017. URL: https://openreview.net/forum?id=BybtVK9lg.

[167] Mathieu Emile “Continuous Hierarchical Representations with Poincaré Variational Auto-Encoders”. In: Advances in Neural Information Processing Systems. Vol. 32. Curran Associates, Inc., 2019. URL: https://proceedings.neurips.cc/paper_files/paper/2019/file/0ec04cb3912c4f08874dd03716f80df1-Paper.pdf.

[168] Davidson Tim R. Hyperspherical Variational Auto-Encoders. 2022. arXiv: 1804.00891 [stat.ML].

[169] Kim Juno “t3-Variational Autoencoder: Learning Heavy-tailed Data with Student’s t and Power Divergence”. In: The Twelfth International Conference on Learning Representations. 2024. URL: https://openreview.net/forum?id=RzNlECeoOB.

[170] Goris Robbe LT “Partitioning neuronal variability”. In: Nature neuroscience 17.6 (2014), pp. 858–865. DOI: 10.1038/nn.3711. 24777419 PMC4135707

[171] Cybenko George. “Approximation by superpositions of a sigmoidal function”. In: Mathematics of control, signals and systems 2.4 (1989), pp. 303–314. DOI: 10.1007/BF02551274.

[172] Cremer Chris “Inference Suboptimality in Variational Autoencoders”. In: Proceedings of the 35th International Conference on Machine Learning. Ed. by Jennifer Dy and Andreas Krause. Vol. 80. Proceedings of Machine Learning Research. PMLR, July 2018, pp. 1078–1086. URL: https://proceedings.mlr.press/v80/cremer18a.html.

[173] O’Neill Charleset al. Compute Optimal Inference and Provable Amortisation Gap in Sparse Autoencoders. 2025. arXiv: 2411.13117 [cs.LG].

[174] Krishnan Rahul “On the challenges of learning with inference networks on sparse, high-dimensional data”. In: Proceedings of the Twenty-First International Conference on Artificial Intelligence and Statistics. Ed. by Amos Storkey and Fernando Perez-Cruz. Vol. 84. Proceedings of Machine Learning Research. PMLR, Apr. 2018, pp. 143–151. URL: https://proceedings.mlr.press/v84/krishnan18a.html.

[175] Hoffman Matthew D. “Stochastic Variational Inference”. In: Journal of Machine Learning Research 14.40 (2013), pp. 1303–1347. URL: http://jmlr.org/papers/v14/hoffman13a.html.

[176] Dempster Arthur P “Maximum likelihood from incomplete data via the EM algorithm”. In: Journal of the royal statistical society: series B (methodological) 39.1 (1977), pp. 1–22. DOI: 10.1111/j.2517-6161.1977.tb01600.x.

[177] Bishop Christopher M.. Pattern Recognition and Machine Learning. New York: Springer, 2006. URL: https://www.microsoft.com/en-us/research/wp-content/uploads/2006/01/Bishop-Pattern-Recognition-and-Machine-Learning-2006.pdf.

[178] Wainwright Martin J. and Jordan Michael I.. “Graphical Models, Exponential Families, and Variational Inference”. In: Foundations and Trends^®^ in Machine Learning 1.1-2 (2008), pp. 1–305. ISSN: 1935–8237. DOI: 10.1561/2200000001. URL: http://dx.doi.org/10.1561/2200000001.

[179] Minka Thomas P.. Expectation Propagation for approximate Bayesian inference. 2013. arXiv: 1301.2294 [cs.AI].

[180] Hospedales Timothy “Meta-learning in neural networks: A survey”. In: IEEE transactions on pattern analysis and machine intelligence 44.9 (2021), pp. 5149–5169. DOI: 10.1109/TPAMI.2021.3079209. 33974543

[181] Teich Malvin C. “Fractal character of the auditory neural spike train”. In: IEEE Transactions on Biomedical Engineering 36.1 (1989), pp. 150–160. DOI: 10.1109/10.16460. 2921061

[182] Shadlen Michael Nand Newsome William T. “The variable discharge of cortical neurons: implications for connectivity, computation, and information coding”. In: Journal of neuroscience 18.10 (1998), pp. 3870–3896. DOI: 10.1523/JNEUROSCI.18-10-03870.1998. 9570816 PMC6793166

[183] Rieke Fred Spikes: exploring the neural code. MIT press, 1999. URL: https://mitpress.mit.edu/9780262181747/spikes/.

[184] Truccolo Wilson “A point process framework for relating neural spiking activity to spiking history, neural ensemble, and extrinsic covariate effects”. In: Journal of neurophysiology 93.2 (2005), pp. 1074–1089. DOI: 10.1152/jn.00697.2004. 15356183

[185] Mainen Zachary Fand Sejnowski Terrence J. “Reliability of spike timing in neocortical neurons”. In: Science 268.5216 (1995), pp. 1503–1506. DOI: 10.1126/science.7770778. 7770778

[186] Butts Daniel A “Nonlinear computations shaping temporal processing of precortical vision”. In: Journal of Neurophysiology 116.3 (2016), pp. 1344–1357. DOI: 10.1152/jn.00878.2015. PMC5040380 27334959

[187] Weber Alison I. and Pillow Jonathan W.. “Capturing the Dynamical Repertoire of Single Neurons with Generalized Linear Models”. In: Neural Computation 29.12 (Dec. 2017), pp. 3260–3289. ISSN: 0899–7667. DOI: 10.1162/neco_a_01021. 28957020

[188] Landau L.D. and Lifshitz E.M.. The Classical Theory of Fields. 4th. Vol. 2. Course of Theoretical Physics. Pergamon Press, 1975. URL: https://perso.crans.org/sylvainrey/Biblio%20Physique/Physique/%C3%89lectromagn%C3%A9tisme/%5BLandau%2C%20Lifshitz%5D%2002%20The%20Classical%20Theory%20of%20Fields.pdf.

[189] Weinberg Steven. “Effective field theory, past and future”. In: International Journal of Modern Physics A 31.06 (2016), p. 1630007. DOI: 10.1142/S0217751X16300076.

[190] Schwichtenberg Jakob. No-Nonsense Quantum Field Theory: A Student-Friendly Introduction. No-Nonsense Books, 2020. ISBN: 9783948763015. URL: http://www.stat.ucla.edu/~ywu/QFT0.pdf.

[191] Schwichtenberg Jakob. Physics from symmetry. Springer, 2018. DOI: 10.1007/978-3-319-66631-0.

[192] Adrian Edgar Douglasand Zotterman Yngve. “The impulses produced by sensory nerve-endings: Part II. The response of a Single End-Organ”. In: The Journal of Physiology (1926), pp. 151–71. DOI: 10.1113/jphysiol.1926.sp002281. PMC1514782 16993780

[193] Amari Shun-ichi. Information geometry and its applications. Vol. 194. Springer, 2016. DOI: 10.1007/978-4-431-55978-8.

[194] Sherman S. M. and Guillery R. W.. “Functional organization of thalamocortical relays”. In: Journal of Neurophysiology 76.3 (1996). pp. 1367–1395. DOI: 10.1152/jn.1996.76.3.1367. 8890259

[195] Adams P. “The role of the thalamus in the flow of information to the cortex”. In: Philosophical Transactions of the Royal Society of London. Series B: Biological Sciences 357.1428 (2002), pp. 1695–1708. DOI: 10.1098/rstb.2002.1161. PMC1693087 12626004

[196] Blakemore Colin and Tobin Elisabeth A. “Lateral inhibition between orientation detectors in the cat’s visual cortex”. In: Experimental brain research 15 (1972), pp. 439–440. DOI: 10.1007/BF00234129. 5079475

[197] Szlam Arthur “Structured sparse coding via lateral inhibition”. In: Advances in Neural Information Processing Systems. Ed. by J. Shawe-Taylor et al. Vol. 24. Curran Associates, Inc., 2011. URL: https://proceedings.neurips.cc/paper_files/paper/2011/file/fae0b27c451c728867a567e8c1bb4e53-Paper.pdf.

[198] Adesnik Hillel and Scanziani Massimo. “Lateral competition for cortical space by layer-specific horizontal circuits”. In: Nature 464.7292 (2010), pp. 1155–1160. DOI: 10.1038/nature08935. PMC2908490 20414303

[199] Luo Liqun. “Architectures of neuronal circuits”. In: Science 373.6559 (2021). DOI: 10.1126/science.abg7285. PMC8916593 34516844

[200] Del Rosario Joseph “Lateral inhibition in V1 controls neural and perceptual contrast sensitivity”. In: Nature Neuroscience (2025), pp. 1–12. DOI: 10.1038/s41593-025-01888-4. PMC12743187 40033123

[201] Benda Jan and Herz Andreas V. M.. “A Universal Model for Spike-Frequency Adaptation”. In: Neural Computation 15.11 (Nov. 2003), pp. 2523–2564. ISSN: 0899–7667. DOI: 10.1162/089976603322385063. 14577853

[202] Hobson Arthur. “A new theorem of information theory”. In: Journal of Statistical Physics 1 (1969), pp. 383–391. DOI: 10.1007/BF01106578.

[203] Koch Christof. Biophysics of Computation: Information Processing in Single Neurons. Oxford University Press, Nov. 1998. ISBN: 9780195104912. DOI: 10.1093/oso/9780195104912.001.0001.

[204] He Kaiming “Deep Residual Learning for Image Recognition”. In: Proceedings of the IEEE Conference on Computer Vision and Pattern Recognition (CVPR). June 2016. URL: https://openaccess.thecvf.com/content_cvpr_2016/html/He_Deep_Residual_Learning_CVPR_2016_paper.html.

[205] Bricken Trenton “Emergence of Sparse Representations from Noise”. In: Proceedings of the 40th International Conference on Machine Learning. Ed. by Andreas Krause et al. Vol. 202. Proceedings of Machine Learning Research. PMLR, July 2023, pp. 3148–3191. URL: https://proceedings.mlr.press/v202/bricken23a.html.

[206] Yuille Alan and Kersten Daniel. “Vision as Bayesian inference: analysis by synthesis?” In: Trends in Cognitive Sciences 10.7 (2006), pp. 301–308. DOI: 10.1016/j.tics.2006.05. 002. 16784882

[207] Teti Michael. LCA-PyTorch. [Computer Software] https://doi.org/10.11578/dc.20230728.4. June 2023. DOI: 10.11578/dc.20230728.4.

[208] Kingma Diederik Pand Ba Jimmy. “Adam: A method for stochastic optimization”. In: (2014). arXiv: 1412.6980 [cs.LG].

[209] Loshchilov Ilya and Hutter Frank. “SGDR: Stochastic Gradient Descent with Warm Restarts”. In: International Conference on Learning Representations. 2017. URL: https://openreview.net/forum?id=Skq89Scxx.

[210] Bowman Samuel R. “Generating Sentences from a Continuous Space”. In: Proceedings of the 20th SIGNLL Conference on Computational Natural Language Learning. Berlin, Germany: Association for Computational Linguistics, Aug. 2016, pp. 10–21. DOI: 10.18653/v1/K16-1002.

[211] Fu Hao “Cyclical Annealing Schedule: A Simple Approach to Mitigating KL Vanishing”. In: Proceedings of the 2019 Conference of the North American Chapter of the Association for Computational Linguistics: Human Language Technologies, Volume 1 (Long and Short Papers). Minneapolis, Minnesota: Association for Computational Linguistics, June 2019, pp. 240–250. DOI: 10.18653/v1/N19-1021.

[212] Lecun Y. “Gradient-based learning applied to document recognition”. In: Proceedings of the IEEE 86.11 (1998), pp. 2278–2324. DOI: 10.1109/5.726791.

[213] Zhu Mengchen and Rozell Christopher J. “Visual nonclassical receptive field effects emerge from sparse coding in a dynamical system”. In: PLoS computational biology 9.8 (2013), e1003191. DOI: 10.1371/journal.pcbi.1003191. PMC3757072 24009491

[214] Carandini Matteo and Heeger David J. “Normalization as a canonical neural computation”. In: Nature reviews neuroscience 13.1 (2012), pp. 51–62. DOI: 10.1038/nrn3136. PMC3273486 22108672

[215] Cohen Gregory “EMNIST: Extending MNIST to handwritten letters”. In: 2017 international joint conference on neural networks (IJCNN). IEEE. 2017, pp. 2921–2926. DOI: 10.1109/IJCNN.2017.7966217.

[216] Lake Brenden M “Human-level concept learning through probabilistic program induction”. In: Science 350.6266 (2015), pp. 1332–1338. DOI: 10.1126/science.aab3050. 26659050

[217] Chrabaszcz Patryk A Downsampled Variant of ImageNet as an Alternative to the CIFAR datasets. 2017. arXiv: 1707.08819 [cs.CV].

[218] Ghifary Muhammad “Domain Generalization for Object Recognition With Multi-Task Autoencoders”. In: Proceedings of the IEEE International Conference on Computer Vision (ICCV). Dec. 2015. URL: https://openaccess.thecvf.com/content_iccv_2015/html/Ghifary_Domain_Generalization_for_ICCV_2015_paper.html.

[219] Lee Honglak “Sparse deep belief net model for visual area V2”. In: Advances in neural information processing systems 20 (2007). URL: https://papers.nips.cc/paper_files/paper/2007/file/4daa3db355ef2b0e64b472968cb70f0d-Paper.pdf.

[220] Rombach Robin “High-Resolution Image Synthesis With Latent Diffusion Models”. In: Proceedings of the IEEE/CVF Conference on Computer Vision and Pattern Recognition (CVPR). June 2022, pp. 10684–10695. URL: https://openaccess.thecvf.com/content/CVPR2022/html/Rombach_High-Resolution_Image_Synthesis_With_Latent_Diffusion_Models_CVPR_2022_paper.

[221] Yang Ling Diffusion Models: A Comprehensive Survey of Methods and Applications. 2024. arXiv: 2209.00796 [cs.LG]. URL: https://arxiv.org/abs/2209.00796.

[222] Martens James. “New Insights and Perspectives on the Natural Gradient Method”. In: Journal of Machine Learning Research 21.146 (2020), pp. 1–76. URL: http://jmlr.org/papers/v21/17-678.html.

[223] Adrian Edgar. “The activity of the nerve fibres”. In: Nobel lecture (1932). URL: https://www.nobelprize.org/prizes/medicine/1932/adrian/lecture/.

[224] Perkel Donald Hand Bullock Theodore H. “Neural coding”. In: Neurosciences Research Program Bulletin (1968). URL: https://ntrs.nasa.gov/citations/19690022317.

[225] Zohary Ehud “Correlated neuronal discharge rate and its implications for psychophysical performance”. In: Nature 370.6485 (1994), pp. 140–143. DOI: 10.1038/370140a0. 8022482

[226] Sze Vivienne “Efficient processing of deep neural networks: A tutorial and survey”. In: Proceedings of the IEEE 105.12 (2017), pp. 2295–2329. DOI: 10.1109/JPROC.2017.2761740.

[227] Chen Yu-Hsin “Eyeriss: An energy-efficient reconfigurable accelerator for deep convolutional neural networks”. In: IEEE journal of solid-state circuits 52.1 (2016), pp. 127–138. DOI: 10.1109/JSSC.2016.2616357.

[228] Du Xuexing “A generalized Spiking Locally Competitive Algorithm for multiple optimization problems”. In: Neurocomputing 624 (2025), p. 129392. ISSN: 0925–2312. DOI: 10.1016/j.neucom.2025.129392.

[229] Hastings W. K.. “Monte Carlo sampling methods using Markov chains and their applications”. In: Biometrika 57.1 (Apr. 1970), pp. 97–109. ISSN: 0006–3444. DOI: 10.1093/biomet/57.1.97.

[230] Gelfand Alan E. and Smith Adrian F. M. and. “Sampling-Based Approaches to Calculating Marginal Densities”. In: Journal of the American Statistical Association 85.410 (1990), pp. 398–409. DOI: 10.1080/01621459.1990.10476213.

[231] Brooks Steve Handbook of markov chain monte carlo. CRC press, 2011. DOI: 10.1201/b10905.

[232] Naesseth Christian A. “Elements of Sequential Monte Carlo”. In: Foundations and Trends^®^ in Machine Learning 12.3 (2019), pp. 307–392. ISSN: 1935–8237. DOI: 10.1561/2200000074. URL: http://dx.doi.org/10.1561/2200000074.

[233] Fiser József “Statistically optimal perception and learning: from behavior to neural representations”. In: Trends in cognitive sciences 14.3 (2010), pp. 119–130. DOI: 10.1016/j.tics.2010.01.003. PMC2939867 20153683

[234] Buesing Lars “Neural Dynamics as Sampling: A Model for Stochastic Computation in Recurrent Networks of Spiking Neurons”. In: PLOS Computational Biology 7.11 (Nov. 2011), pp. 1–22. DOI: 10.1371/journal.pcbi.1002211. PMC3207943 22096452

[235] Haefner Ralf M “Perceptual decision-making as probabilistic inference by neural sampling”. In: Neuron 90.3 (2016), pp. 649–660. DOI: 10.1016/j.neuron.2016.03.020. 27146267

[236] Echeveste Rodrigo “Cortical-like dynamics in recurrent circuits optimized for sampling-based probabilistic inference”. In: Nature neuroscience 23.9 (2020), pp. 1138–1149. DOI: 10.1038/s41593-020-0671-1. 32778794 PMC7610392

[237] Buxó Camille Rullánand Savin Cristina. “A sampling-based circuit for optimal decision making”. In: Advances in Neural Information Processing Systems. Vol. 34. Curran Associates, Inc., 2021, pp. 14163–14175. URL: https://proceedings.neurips.cc/paper/2021/hash/76444b3132fda0e2aca778051d776f1c-Abstract.html.

[238] Savin Cristina and Deneve Sophie. “Spatio-temporal Representations of Uncertainty in Spiking Neural Networks”. In: Advances in Neural Information Processing Systems. Ed. by Z. Ghahramani Vol. 27. Curran Associates, Inc., 2014. URL: https://proceedings.neurips.cc/paper_files/paper/2014/file/02a12643ae21d984b93c9df82a9d2152-Paper.pdf.

[239] Festa Dylan “Neuronal variability reflects probabilistic inference tuned to natural image statistics”. In: Nature communications 12.1 (2021), p. 3635. DOI: 10.1038/s41467-021-23838-x. PMC8206154 34131142

[240] Shrinivasan Suhas “Taking the neural sampling code very seriously: A data-driven approach for evaluating generative models of the visual system”. In: Advances in Neural Information Processing Systems. Ed. by Oh A.et al. Vol. 36. Curran Associates, Inc., 2023, pp. 21945–21959. URL: https://proceedings.neurips.cc/paper_files/paper/2023/hash/458d9f2dd5c7565af60143630dc62f10-Abstract-Conference.html.

[241] Zhang Wen-Hao “Sampling-based Bayesian inference in recurrent circuits of stochastic spiking neurons”. In: Nature communications 14.1 (2023), p. 7074. DOI: 10.1038/s41467-023-41743-3. PMC10625605 37925497

[242] Haefner Ralf M. How does the brain compute with probabilities? 2024. arXiv: 2409. 02709 [q-bio.NC].

[243] Ahmadian Yashar “Analysis of the Stabilized Supralinear Network”. In: Neural Computation 25.8 (Aug. 2013), pp. 1994–2037. ISSN: 0899–7667. DOI: 10.1162/NECO_a_00472. PMC4026108 23663149

[244] Hennequin Guillaume “The dynamical regime of sensory cortex: stable dynamics around a single stimulus-tuned attractor account for patterns of noise variability”. In: Neuron 98.4 (2018), pp. 846–860. DOI: 10.1016/j.neuron.2018.04.017. PMC5971207 29772203

[245] Ma Yi-An “A Complete Recipe for Stochastic Gradient MCMC”. In: Advances in Neural Information Processing Systems. Ed. by Cortes C.et al. Vol. 28. Curran Associates, Inc., 2015. URL: https://proceedings.neurips.cc/paper_files/paper/2015/hash/9a4400501febb2a95e79248486a5f6d3-Abstract.html.

[246] Salimans Tim “Markov Chain Monte Carlo and Variational Inference: Bridging the Gap”. In: Proceedings of the 32nd International Conference on Machine Learning. Ed. by Francis Bach and David Blei. Vol. 37. Proceedings of Machine Learning Research. Lille, France: PMLR, July 2015, pp. 1218–1226. URL: https://proceedings.mlr.press/v37/salimans15.html.

[247] Lange Richard D. “Interpolating between sampling and variational inference with infinite stochastic mixtures”. In: Proceedings of the Thirty-Eighth Conference on Uncertainty in Artificial Intelligence. Ed. by James Cussens and Kun Zhang. Vol. 180. Proceedings of Machine Learning Research. PMLR, Aug. 2022, pp. 1063–1073. URL: https://proceedings.mlr.press/v180/lange22a.html.

[248] Wei Jason “Chain-of-Thought Prompting Elicits Reasoning in Large Language Models”. In: Advances in Neural Information Processing Systems. Ed. by Koyejo S.et al. Vol. 35. Curran Associates, Inc., 2022, pp. 24824–24837. URL: https://proceedings.neurips.cc/paper_files/paper/2022/hash/9d5609613524ecf4f15af0f7b31abca4-Abstract-Conference.html.

[249] Yao Shunyu “Tree of Thoughts: Deliberate Problem Solving with Large Language Models”. In: Advances in Neural Information Processing Systems. Ed. by Oh A.et al. Vol. 36. Curran Associates, Inc., 2023, pp. 11809–11822. URL: https://proceedings.neurips.cc/paper_files/paper/2023/hash/271db9922b8d1f4dd7aaef84ed5ac703-Abstract-Conference.html.

[250] Liao Isaac and Gu Albert. ARC-AGI Without Pretraining. Mar. 2025. URL: https://iliao2345.github.io/blog_posts/arc_agi_without_pretraining/arc_agi_without_pretraining.html.

[251] Chollet Francois ARC Prize 2024: Technical Report. 2025. arXiv: 2412.04604 [cs.AI].

[252] Dao Tri “FlashAttention: Fast and Memory-Efficient Exact Attention with IO-Awareness”. In: Advances in Neural Information Processing Systems. Ed. by Koyejo S.et al. Vol. 35. Curran Associates, Inc., 2022, pp. 16344–16359. URL: https://proceedings.neurips.cc/paper_files/paper/2022/hash/67d57c32e20fd0a7a302cb81d36e40d5-Abstract-Conference.html.

[253] Gu Albert and Dao Tri. “Mamba: Linear-Time Sequence Modeling with Selective State Spaces”. In: First Conference on Language Modeling. 2024. URL: https://openreview.net/forum?id=tEYskw1VY2.

[254] Schmied Thomas A Large Recurrent Action Model: xLSTM enables Fast Inference for Robotics Tasks. 2025. arXiv: 2410.22391 [cs.LG].

[255] Pope Reiner “Efficiently Scaling Transformer Inference”. In: Proceedings of Machine Learning and Systems. Ed. by Song D.et al. Vol. 5. Curan, 2023, pp. 606–624. URL: https://proceedings.mlsys.org/paper_files/paper/2023/hash/c4be71ab8d24cdfb45e3d06dbfca2780-Abstract-mlsys2023.html.

[256] Chen Charlie Accelerating Large Language Model Decoding with Speculative Sampling. 2023. arXiv: 2302.01318 [cs.CL].

[257] Grossberg Stephen. “Competitive learning: From interactive activation to adaptive resonance”. In: Cognitive Science 11.1 (1987), pp. 23–63. ISSN: 0364–0213. DOI: 10.1016/S0364-0213(87)80025-3.

[258] Bellec Guillaume “A solution to the learning dilemma for recurrent networks of spiking neurons”. In: Nature communications 11.1 (2020), p. 3625. DOI: 10.1038/s41467-020-17236-y. PMC7367848 32681001

[259] Payeur Alexandre “Burst-dependent synaptic plasticity can coordinate learning in hierarchical circuits”. In: Nature neuroscience 24.7 (2021), pp. 1010–1019. DOI: 10.1038/s41593-021-00857-x. 33986551

[260] Whittington James C. R. and Bogacz Rafal. “An Approximation of the Error Backpropagation Algorithm in a Predictive Coding Network with Local Hebbian Synaptic Plasticity”. In: Neural Computation 29.5 (May 2017), pp. 1229–1262. ISSN: 0899–7667. DOI: 10.1162/NECO_a_00949. PMC5467749 28333583

[261] Song Sen “Highly Nonrandom Features of Synaptic Connectivity in Local Cortical Circuits”. In: PLOS Biology 3.3 (Mar. 2005). DOI: 10.1371/journal.pbio.0030068. PMC1054880 15737062

[262] Whittington James CRand Bogacz Rafal. “Theories of error back-propagation in the brain”. In: Trends in cognitive sciences 23.3 (2019), pp. 235–250. DOI: 10.1016/j.tics.2018.12.005. PMC6382460 30704969

[263] Lillicrap Timothy P “Backpropagation and the brain”. In: Nature Reviews Neuroscience 21.6 (2020), pp. 335–346. DOI: 10.1038/s41583-020-0277-3. 32303713

[264] Dobs Katharina “Brain-like functional specialization emerges spontaneously in deep neural networks”. In: Science Advances 8.11 (2022), eabl8913. DOI: 10.1126/sciadv.abl8913. PMC8926347 35294241

[265] Gerstner Wulfram “Eligibility traces and plasticity on behavioral time scales: experimental support of neohebbian three-factor learning rules”. In: Frontiers in neural circuits 12 (2018), p. 53. DOI: 10.3389/fncir.2018.00053. PMC6079224 30108488

[266] Lillicrap Timothy P “Random synaptic feedback weights support error backpropagation for deep learning”. In: Nature communications 7.1 (2016), p. 13276. DOI: 10.1038/ncomms13276. PMC5105169 27824044

[267] Toosi Tahereh and Issa Elias. “Brain-like Flexible Visual Inference by Harnessing Feedback Feedforward Alignment”. In: Advances in Neural Information Processing Systems. Ed. by Oh A. Vol. 36. Curran Associates, Inc., 2023, pp. 56979–56997. URL: https://proceedings.neurips.cc/paper_files/paper/2023/file/b29ec434e049fb96f3c4245a405ee976-Paper-Conference.pdf. PMC11567678 39553924

[268] Bastos Andre M “Canonical microcircuits for predictive coding”. In: Neuron 76.4 (2012), pp. 695–711. DOI: 10.1016/j.neuron.2012.10.038. PMC3777738 23177956

[269] Dimakou Anastasia “The predictive nature of spontaneous brain activity across scales and species”. In: Neuron (2025). DOI: 10.1016/j.neuron.2025.02.009. 40101720

[270] Liu Belle “Predictive encoding of motion begins in the primate retina”. In: Nature neuroscience 24.9 (2021), pp. 1280–1291. DOI: 10.1038/s41593-021-00899-1. 34341586 PMC8728393

[271] Keller Georg Band Mrsic-Flogel Thomas D. “Predictive processing: a canonical cortical computation”. In: Neuron 100.2 (2018). DOI: 10.1016/j.neuron.2018.10.003. PMC6400266 30359606

[272] Chen Tsai-Wen “A Map of Anticipatory Activity in Mouse Motor Cortex”. In: Neuron 94 (2017), 866–879.e4. DOI: 10.1016/j.neuron.2017.05.005. 28521137

[273] Jiang Linxing Prestonand Rao Rajesh P. N.. “Dynamic predictive coding: A model of hierarchical sequence learning and prediction in the neocortex”. In: PLOS Computational Biology 20.2 (Feb. 2024), pp. 1–30. DOI: 10.1371/journal.pcbi.1011801. PMC10880975 38330098

[274] Ha David “HyperNetworks”. In: International Conference on Learning Representations. 2017. URL: https://openreview.net/forum?id=rkpACe1lx.

[275] Fiquet Pierre-Étienne Hand Simoncelli Eero P. “A polar prediction model for learning to represent visual transformations”. In: Thirty-seventh Conference on Neural Information Processing Systems. 2023. URL: https://openreview.net/forum?id=hyPUZX03Ks.

[276] Hénaff Olivier J “Perceptual straightening of natural videos”. In: Nature neuroscience 22.6 (2019), pp. 984–991. DOI: 10.1038/s41593-019-0377-4. 31036946

[277] Lange Richard D “Bayesian encoding and decoding as distinct perspectives on neural coding”. In: Nature Neuroscience 26.12 (2023), pp. 2063–2072. DOI: 10.1038/s41593-023-01458-6. 37996525 PMC11003438

[278] Bechtel William and Bich Leonardo. “Grounding cognition: heterarchical control mechanisms in biology”. In: Philosophical Transactions of the Royal Society B: Biological Sciences 376.1820 (2021), p. 20190751. DOI: 10.1098/rstb.2019.0751. PMC7934967 33487110

[279] Salvatori Tommaso “Learning on Arbitrary Graph Topologies via Predictive Coding”. In: Advances in Neural Information Processing Systems. Ed. by Koyejo S.et al. Vol. 35. Curran Associates, Inc., 2022, pp. 38232–38244. URL: https://proceedings.neurips.cc/paper_files/paper/2022/hash/f9f54762cbb4fe4dbffdd4f792c31221-Abstract-Conference.html. PMC7614467 37090087

[280] Boutin Victor “Pooling strategies in V1 can account for the functional and structural diversity across species”. In: PLOS Computational Biology 18.7 (July 2022), pp. 1–21. DOI: 10.1371/journal.pcbi.1010270. PMC9345491 35862423

[281] Fukushima Kunihiko. “Neocognitron: A self-organizing neural network model for a mechanism of pattern recognition unaffected by shift in position”. In: Biological cybernetics 36.4 (1980), pp. 193–202. DOI: 10.1007/BF00344251. 7370364

[282] Izhikevich Eugene M.. “Polychronization: Computation with Spikes”. In: Neural Computation 18.2 (Feb. 2006), pp. 245–282. ISSN: 0899–7667. DOI: 10.1162/089976606775093882. 16378515

[283] Rullán Buxó Camille E. and Pillow Jonathan W.. “Poisson balanced spiking networks”. In: PLOS Computational Biology 16.11 (Nov. 2020), pp. 1–27. DOI: 10.1371/journal.pcbi.1008261. URL: https://doi.org/10.1371/journal.pcbi.1008261. PMC7717583 33216741

[284] Chen Yusi “Predictive sequence learning in the hippocampal formation”. In: Neuron 112.15 (2024), pp. 2645–2658. DOI: 10.1016/j.neuron.2024.05.024. 38917804

[285] Anderson Keller T. A Spacetime Perspective on Dynamical Computation in Neural Information Processing Systems. 2024. arXiv: 2409.13669 [q-bio.NC].

[286] Yamins Daniel LK “Performance-optimized hierarchical models predict neural responses in higher visual cortex”. In: Proceedings of the National Academy of Sciences 111.23 (2014), pp. 8619–8624. DOI: 10.1073/pnas.1403112111. PMC4060707 24812127

[287] Carandini Matteo “Do we know what the early visual system does?” In: Journal of Neuroscience 25.46 (2005), pp. 10577–10597. DOI: 10.1523/JNEUROSCI.3726-05.2005. 16291931 PMC6725861

[288] Michael C.-K. Wu “COMPLETE FUNCTIONAL CHARACTERIZATION OF SENSORY NEURONS BY SYSTEM IDENTIFICATION”. In: Annual Review of Neuroscience 29.Volume 29, 2006 (2006), pp. 477–505. ISSN: 1545–4126. DOI: 10.1146/annurev.neuro.29.051605.113024. 16776594

[289] Olshausen Bruno A. and Field David J.. “How Close Are We to Understanding V1?” In: Neural Computation 17.8 (Aug. 2005), pp. 1665–1699. ISSN: 0899–7667. DOI: 10.1162/0899766054026639. 15969914

[290] Kanwisher Nancy “Using artificial neural networks to ask ‘why’ questions of minds and brains”. In: Trends in Neurosciences (2023). DOI: 10.1016/j.tins.2022.12.008. 36658072

[291] Sinz Fabian H “Engineering a less artificial intelligence”. In: Neuron 103.6 (2019), pp. 967–979. DOI: 10.1016/j.neuron.2019.08.034. 31557461

